# Supplementary material for: Predicting and designing therapeutics against the Nipah virus
Source: PLoS Negl Trop Dis. 2019 Dec 12;13(12):e0007419. doi: 10.1371/journal.pntd.0007419 (PMC6907750; doi:10.1371/journal.pntd.0007419)
Supplement: S1 Table — The best model predicted by I-TASSER (based on their C-Score) have their Normalized DOPE scores and C-scores in bold. TM-scores and RMSDs are only calculated for the best models. L protein was divided into three domains, indicated by their residue numbers in parentheses, and modeled separately. (DOCX) [file pntd.0007419.s001.docx]

| **Protein** | **Normalized DOPE** | **C-score** | **Predicted TM-score^$^** | **Predicted RMSD** |
| --- | --- | --- | --- | --- |
| V | **2.09**, 1.70, 1.99, 1.23, 1.27 | **-0.79**, -1.82, -0.32, -3.56, -2.69 | 0.61 | 8.9 |
| W | **1.45**, 0.77, 0.75, 1.56, 0.80 | **-1.42**, -1.73, -3.07, -4.34, -3.30 | 0.54 | 10.4 |
| C | **0.49**, -0.08, -1.53, -0.33, -0.88 | **-3.68**, -3.67, -3.29, -4.16, -4.09 | 0.31 | 13.6 |
| L^#^ (14 - 1177) | **0.29**, 0.31, -0.04, 0.01, -0.16 | **0.07**, -0.30, -1.87, -1.05, -0.83 | 0.72 | 9.1 |
| L^#^ (1191 - 1435) | **0.52** | **1.1** | 0.86 | 3.6 |
| L^#^ (1553 - 1859) | **0.21**, 0.82, 0.95, 2.69, 0.44 | **-2.61**, -4.24, -4.52, -4.76, -5.00 | 0.41 | 12.4 |

^#^The protein was built domain wise because I-TASSER has a maximum size limit of 1500 residues.

^$^Although models built for V, W proteins and two of the Polymerase L domains had a TM-scores greater than 0.5, none of these models had a Normalized DOPE score less than or equal to zero and therefore were not used further in the study.
