# Supplementary material for: Predicting and designing therapeutics against the Nipah virus
Source: PLoS Negl Trop Dis. 2019 Dec 12;13(12):e0007419. doi: 10.1371/journal.pntd.0007419 (PMC6907750; doi:10.1371/journal.pntd.0007419)
Supplement: S2 Table — (DOCX) [file pntd.0007419.s002.docx]

| **Run** | **Energy (kJ/mol)** | | **Protein-peptide distance (nm)** | | | **Number of Hydrogen Bonds** | | | **RMSD (nm)** | | **Binding energies (kJ/mol)** | |
| --- | --- | --- | --- | --- | --- | --- | --- | --- | --- | --- | --- | --- |
|  | **Mean** | **SD** | | **Mean** | **SD** | | **Mean** | **SD** | **Mean** | **SD** | **Mean** | **SD** |
| 1 | -510162 | 1093 | | 1.93 | 0.05 | | 5.54 | 1.48 | 0.23 | 0.04 | -102.5 | 9.6 |
| 2 | -510194 | 1084 | | 1.89 | 0.05 | | 4.99 | 1.74 | 0.28 | 0.04 | -117.8 | 9.2 |
| 3 | -510179 | 1090 | | 1.87 | 0.06 | | 4.62 | 1.32 | 0.26 | 0.04 | -102.9 | 9.7 |
| **Mean** | **-510178** |  | | **1.90** |  | | **5.05** |  | **0.26** |  | **-107.7** |  |
