# Supplementary material for: Predicting and designing therapeutics against the Nipah virus
Source: PLoS Negl Trop Dis. 2019 Dec 12;13(12):e0007419. doi: 10.1371/journal.pntd.0007419 (PMC6907750; doi:10.1371/journal.pntd.0007419)
Supplement: S3 Table — (DOCX) [file pntd.0007419.s003.docx]

| **Index** | **Hydrogen Bond Partner** | **Run 1 (%)** | **Run 2 (%)** | **Run 3 (%)** |
| --- | --- | --- | --- | --- |
| 0 | 56ILE.D-26THR.E | 1 | 1 | 1 |
| 1 | 56ILE.D-28GLU.C | 41.6 | 55.4 | 59.4 |
| 2 | 59SER.D-19ALA.E | 100 | 1 | 12.9 |
| 3 | 59SER.D-24GLN.C | 77.2 | 75.2 | 68.3 |
| 4 | 60LYS.D-24GLN.C | 5.9 | 18.8 | 17.8 |
| 5 | 65LYS.D-15SER.E | 5.9 | 22.8 | 5 |
| 6 | 65LYS.D-18GLU.E | 2 | 42.6 | 1 |
| 7 | 66ALA.D-12SER.E | 97 | 64.4 | 97 |
| 8 | 67GLN.D-14GLU.C | 2 | 12.9 | 1 |
| 9 | 67GLN.D-17ASN.C | 36.6 | 2 | 31.7 |
| 10 | 69LEU.D-8LYS.E | 4 | 6.9 | 6.9 |
