# Supplementary material for: Predicting and designing therapeutics against the Nipah virus
Source: PLoS Negl Trop Dis. 2019 Dec 12;13(12):e0007419. doi: 10.1371/journal.pntd.0007419 (PMC6907750; doi:10.1371/journal.pntd.0007419)
Supplement: S4 Table — (DOCX) [file pntd.0007419.s004.docx]

| **Run** | **Energy (kJ/mol)** | | **Protein-peptide distance (nm)** | | | **Hydrogen Bonds** | | | **RMSD (nm)** | | | **Binding energies (kJ/mol)** | |
| --- | --- | --- | --- | --- | --- | --- | --- | --- | --- | --- | --- | --- | --- |
|  | **Mean** | **SD** | **Mean** | **SD** | **Mean** | | **SD** | **Mean** | | **SD** | **Mean** | | **SD** |
| 1 | -1596309 | 1851 | 1.69 | 0.08 | 7.15 | | 2.41 | 0.42 | | 0.08 | -107.9 | | 11.6 |
| 2 | -1596399 | 1868 | 1.89 | 0.09 | 5.73 | | 2.59 | 0.66 | | 0.23 | -121.7 | | 11.9 |
| 3 | -1596287 | 1856 | 1.85 | 0.07 | 6.98 | | 2.29 | 0.56 | | 0.14 | -98.4 | | 12.0 |
| **Mean** | **-1596332** |  | **1.81** |  | **6.62** | |  | **0.55** | |  | **-107.7** | |  |
