# Supplementary material for: Predicting and designing therapeutics against the Nipah virus
Source: PLoS Negl Trop Dis. 2019 Dec 12;13(12):e0007419. doi: 10.1371/journal.pntd.0007419 (PMC6907750; doi:10.1371/journal.pntd.0007419)
Supplement: S5 Table — (DOCX) [file pntd.0007419.s005.docx]

| **Index** | **Hydrogen Bond Partner** | **Run 1 (%)** | **Run 2 (%)** | **Run 3 (%)** |
| --- | --- | --- | --- | --- |
| 0 | 470ARG.B-335PHE.A | 5.0 | 99.0 | 80.2 |
| 1 | 470ARG.B-338TYR.A | 5.9 | 3.0 | 80.2 |
| 2 | 470ARG.B-340ASP.A | 40.6 | 100.0 | 45.5 |
| 3 | 471ARG.B-328GLN.A | 41.6 | 1.0 | 19.8 |
| 4 | 473ALA.B-197ARG.A | 44.6 | 9.9 | 5.9 |
| 5 | 475SER.B-194LEU.A | 1.0 | 5.9 | 1.0 |
| 6 | 475SER.B-195GLU.A | 53.5 | 2.0 | 62.4 |
| 7 | 476THR.B-304ASP.A | 5.9 | 33.7 | 24.8 |
