# Supplementary material for: Predicting and designing therapeutics against the Nipah virus
Source: PLoS Negl Trop Dis. 2019 Dec 12;13(12):e0007419. doi: 10.1371/journal.pntd.0007419 (PMC6907750; doi:10.1371/journal.pntd.0007419)
Supplement: S6 Table — (DOCX) [file pntd.0007419.s006.docx]

| **Run** | **Energy (kJ/mol)** | | **Protein-peptide distance (nm)** | | **RMSD (nm)** | | **Binding energies (kJ/mol)** | |
| --- | --- | --- | --- | --- | --- | --- | --- | --- |
|  | **Mean** | **SD** | **Mean** | **SD** | **Mean** | **SD** | **Mean** | **SD** |
| 1 | -1140578 | 1550 | 1.65 | 0.05 | 0.12 | 0.04 | -107.2 | 10.8 |
| 2 | -1140385 | 1714 | 1.66 | 0.05 | 0.17 | 0.04 | -96.0 | 10.8 |
| 3 | -1140696 | 1708 | 1.64 | 0.03 | 0.13 | 0.03 | -93.9 | 12.2 |
| **Mean** | **-1140553** |  | **1.65** |  | **0.14** |  | **-99.0** |  |
