# Supplementary material for: Predicting and designing therapeutics against the Nipah virus
Source: PLoS Negl Trop Dis. 2019 Dec 12;13(12):e0007419. doi: 10.1371/journal.pntd.0007419 (PMC6907750; doi:10.1371/journal.pntd.0007419)
Supplement: S7 Table — (DOCX) [file pntd.0007419.s007.docx]

| **Run** | **Energy (kJ/mol)** | | **Protein-peptide distance (nm)** | | | **RMSD (nm)** | | **Binding energies (kJ/mol)** | |
| --- | --- | --- | --- | --- | --- | --- | --- | --- | --- |
|  | **Mean** | **SD** | **Mean** | **SD** | **Mean** | | **SD** | **Mean** | **SD** |
| 1 | -1124416 | 1628 | 1.50 | 0.03 | 0.19 | | 0.03 | -61.0 | 12.9 |
| 2 | -1124513 | 1695 | 1.72 | 0.05 | 0.21 | | 0.05 | -58.9 | 11.4 |
| 3 | -1124582 | 1578 | 1.54 | 0.05 | 0.17 | | 0.03 | -65.1 | 12.2 |
| **Mean** | **-1124504** |  | **1.59** |  | **0.19** | |  | **-61.7** |  |
