# Supplementary material for: Predicting and designing therapeutics against the Nipah virus
Source: PLoS Negl Trop Dis. 2019 Dec 12;13(12):e0007419. doi: 10.1371/journal.pntd.0007419 (PMC6907750; doi:10.1371/journal.pntd.0007419)
Supplement: S8 Table — The residue name is followed by the residue number. The chain id has been depicted after the dot. (DOCX) [file pntd.0007419.s008.docx]

| **NiV protein** | **Pocket** | **Pocket lining residue numbers** | **PockDrug** | **CavityPlus** |
| --- | --- | --- | --- | --- |
| Glycoprotein | PG1 | F458.A, W504.A, Q559.A, D219.A, Y280.A, L305.A, Q490.A | 0.43 | Druggable |
|  | PG2 | P500.A, G489.A, R435.A, W479.A, S432.A, E430.A, R344.A, K376.A, F375.A, N378.A, S398.A, P383.A | 0.47 | Less druggable |
|  |  |  |  |  |
| Nucleoprotein | PN1 | S67.A, A65.A, V58.A, I131.A, L128.A, E124.A, R36.A, F38.A, K34.A | 0.99 | Less druggable |
|  | PN2 | K69.A, N219.A, Q223.A, S224.A, L225.A, K229.A, F230.A, I35.A | 0.99 | Undruggable |
|  | PN4 | R218.A, N219.A, S222.A, R228.A, Q319.A, E316.A, I176.A, K178.A | 0.99 | Undruggable |
|  | PN5 | R307.A, Y310.A, V232.A, L314.A, E315.A, S226.A, D94.A, E233.A, L225.A | 0.99 | Undruggable |
|  |  |  |  |  |
| Phosphoprotein | PP1 | T562.B, K559.B, V556.B, N561.C, T562.C, T566.C, E568.C, I567.C | 0.43 | Less druggable |
|  | PP2 | L517.C, E514.C, V516.C, N522.C, D482.B | Pocket not identified | Undruggable |
|  |  |  |  |  |
| Fusion protein | PF2 | V39.B, Y30.B, H29.B, Y432.B, L433.B, N380.B, K40.B | 0.88 | Undruggable |
|  |  |  |  |  |
| Matrix protein | PM1 | E195.A, H238.A, P332.A, Q328.A, L207.A, M236.A, D304.A, M188.A | 0.33 | Druggable |
|  | PM2 | F151.A, K143.A, W141.A, Y62.A, L181.A, Y187.A, M188.A, L274.A, D304.A | 0.96 | Druggable |
|  | PM3 | L312.A, W314.A, L309.A, F235.A, D213.A, M236.A, F266.A | 0.96 | Less druggable |
