# Supplementary material for: Predicting and designing therapeutics against the Nipah virus
Source: PLoS Negl Trop Dis. 2019 Dec 12;13(12):e0007419. doi: 10.1371/journal.pntd.0007419 (PMC6907750; doi:10.1371/journal.pntd.0007419)
Supplement: S9 Table — RMSD_1 –RMSD_5 are the RMSDs of the 5 best Autodock4 poses with the best scoring Dock6.8 pose. The least RMSD is depicted in bold. Cells highlighted in yellow have RMSDs less than 0.15 nm. Pocket number indicates pockets from Autodock4. Some of the Autodock4 pockets have been subdivided by DOCK6.8, which indicates the subsections in each pocket. (DOCX) [file pntd.0007419.s009.docx]

| **Protein name** | **PDB ID** | **Pocket Number** | **Number of selected molecules** | **ZINC ID** | **Rank in DOCK** | **Rank in Autodock4** | **Energy in DOCK6.8** | **Energy in Autodock** | **RMSD_1 (nm)** | **RMSD_2(nm)** | **RMSD_3(nm)** | **RMSD_4(nm)** | **RMSD_5(nm)** |
| --- | --- | --- | --- | --- | --- | --- | --- | --- | --- | --- | --- | --- | --- |
| Glycoprotein | 3D11 | PG1 | 4 | ZINC63411510 | 10 | 60 | -58.1569 | -8.73 | 0.8 | **0.797** | 0.809 | 0.807 | 0.797 |
|  |  | PG1 |  | ZINC93305816 | 145 | 129 | -48.6263 | -8.55 | **0.799** | 0.800 | 0.803 | 0.876 | 0.861 |
|  |  | PG1 |  | ZINC63857604 | 108 | 56 | -49.4608 | -8.76 | **0.695** | 0.698 | 0.697 | **0.695** | 0.697 |
|  |  | PG1 |  | ZINC72264974 | 124 | 120 | -49.0663 | -8.57 | 1.033 | **0.986** | **0.986** | 1.000 | 1.005 |
|  |  |  |  |  |  |  |  |  |  |  |  |  |  |
|  |  | PG2 | 5 | ZINC04580552 | 132 | 7 | -42.6248 | -8.97 | 2.260 | **2.259** | 2.293 | 2.312 | 2.293 |
|  |  | PG2 |  | ZINC23214639 | 124 | 40 | -42.7004 | -8.43 | 2.395 | 2.373 | 2.393 | 2.349 | **2.348** |
|  |  | PG2 |  | ZINC65407076 | 128 | 23 | -42.6745 | -8.64 | **2.209** | 2.211 | 2.232 | 2.225 | 2.246 |
|  |  | PG2 |  | ZINC93655460 | 75 | 128 | -43.7118 | -8.09 | 2.271 | 2.267 | **2.263** | 2.271 | 2.265 |
|  |  | PG2 |  | ZINC94217163 | 60 | 150 | -44.5727 | -8.04 | 2.295 | 2.293 | **2.287** | 2.300 | 2.306 |
|  |  |  |  |  |  |  |  |  |  |  |  |  |  |
| Nucleoprotein | 4CO6 | PN1 | 1 | ZINC34083937 | 138 | 74 | -35.9687 | -8.27 | 1.262 | 1.260 | 1.252 | 1.254 | **1.239** |
|  |  |  |  |  |  |  |  |  |  |  |  |  |  |
|  |  | PN1 | 10 | ZINC42750806 | 48 | 50 | -36.7541 | -8.41 | 0.432 | 0.397 | 0.648 | 0.263 | **0.251** |
|  |  | PN1 |  | ZINC02511792 | 62 | 62 | -36.4557 | -8.33 | 0.333 | 0.336 | 0.332 | **0.32** | 0.334 |
|  |  | PN1 |  | ZINC34083937 | 52 | 73 | -36.6097 | -8.27 | 0.64 | 0.637 | 0.64 | **0.601** | 0.623 |
|  |  | PN1 |  | ZINC05382414 | 68 | 147 | -36.3915 | -8.09 | 0.231 | 0.247 | 0.205 | **0.179** | 0.193 |
|  |  | PN1 |  | ZINC16545537 | 107 | 5 | -35.6182 | -9.08 | 0.147 | **0.145** | 0.147 | 0.160 | 0.158 |
|  |  | PN1 |  | ZINC16954338 | 141 | 15 | -35.1782 | -8.81 | 0.625 | 0.482 | 0.602 | 0.125 | **0.124** |
|  |  | PN1 |  | ZINC63959595 | 25 | 139 | -37.5303 | -8.1 | 0.149 | **0.142** | 0.222 | 0.170 | 0.199 |
|  |  | PN1 |  | ZINC92484162 | 140 | 86 | -35.1959 | -8.23 | 0.605 | 0.608 | 0.602 | **0.600** | 0.606 |
|  |  | PN1 |  | ZINC92722391 | 142 | 40 | -35.1454 | -8.46 | 0.647 | **0.621** | 0.654 | 0.642 | 0.667 |
|  |  | PN1 |  | ZINC92722539 | 125 | 20 | -35.3924 | -8.7 | 0.575 | 0.574 | 0.584 | **0.268** | 0.271 |
|  |  |  |  |  |  |  |  |  |  |  |  |  |  |
|  |  | PN2 | 15 | ZINC94258465 | 24 | 96 | -36.1295 | -7.08 | 0.702 | **0.693** | 0.707 | 0.706 | 0.705 |
|  |  | PN2 |  | ZINC94258558 | 33 | 79 | -35.8008 | -7.17 | 0.081 | **0.074** | 0.074 | 0.096 | 0.076 |
|  |  | PN2 |  | ZINC86657759 | 64 | 54 | -34.8881 | -7.31 | **0.505** | 0.509 | 0.518 | 0.506 | 0.508 |
|  |  | PN2 |  | ZINC73641145 | 6 | 28 | -37.4871 | -7.6 | 0.145 | 0.148 | 0.261 | 0.149 | **0.142** |
|  |  | PN2 |  | ZINC95022396 | 98 | 42 | -34.3319 | -7.43 | 0.672 | 0.674 | 0.688 | 0.68 | **0.661** |
|  |  | PN2 |  | ZINC77262630 | 4 | 50 | -38.6140 | -7.35 | 0.275 | **0.187** | 0.232 | 0.221 | 0.255 |
|  |  | PN2 |  | ZINC72264974 | 91 | 47 | -34.3793 | -7.36 | 1.115 | 1.094 | **1.085** | 1.103 | 1.171 |
|  |  | PN2 |  | ZINC04580552 | 10 | 19 | -36.8562 | -7.79 | 0.375 | 0.376 | 0.286 | **0.272** | 0.442 |
|  |  | PN2 |  | ZINC72107957 | 106 | 8 | -34.1858 | -8.16 | 0.508 | **0.450** | 0.455 | 0.761 | 0.722 |
|  |  | PN2 |  | ZINC85191592 | 11 | 115 | -36.8475 | -7.02 | 0.685 | 0.691 | 0.710 | 0.747 | **0.458** |
|  |  | PN2 |  | ZINC91932783 | 133 | 61 | -34.0056 | -7.26 | 0.173 | 0.173 | **0.072** | 0.167 | 0.503 |
|  |  | PN2 |  | ZINC92349362 | 22 | 125 | -36.1803 | -6.99 | 0.323 | 0.353 | 0.362 | **0.308** | 0.334 |
|  |  | PN2 |  | ZINC94927184 | 2 | 148 | -39.6966 | -6.92 | 0.647 | 0.657 | 0.656 | **0.553** | 0.659 |
|  |  | PN2 |  | ZINC94937158 | 128 | 60 | -34.0391 | -7.28 | **0.675** | 0.699 | 0.733 | 0.700 | 0.720 |
|  |  | PN2 |  | ZINC95355539 | 129 | 94 | -34.0157 | -7.1 | 0.778 | 0.770 | 0.785 | 0.765 | **0.678** |
|  |  |  |  |  |  |  |  |  |  |  |  |  |  |
|  |  | PN2 | 8 | ZINC16755504 | 113 | 137 | -36.6964 | -6.96 | 1.962 | **1.949** | 2.367 | 2.359 | 2.368 |
|  |  | PN2 |  | ZINC72129411 | 122 | 44 | -36.5315 | -7.41 | **1.504** | 1.516 | 1.531 | 1.525 | 1.510 |
|  |  | PN2 |  | ZINC72133204 | 52 | 141 | -37.8067 | -6.94 | 1.843 | **1.833** | 1.843 | 1.836 | 1.844 |
|  |  | PN2 |  | ZINC72388943 | 144 | 35 | -36.2274 | -7.52 | 1.889 | 1.890 | 1.895 | **1.888** | 1.896 |
|  |  | PN2 |  | ZINC91932783 | 136 | 62 | -36.2850 | -7.26 | 2.603 | **2.602** | 2.631 | 2.660 | 2.950 |
|  |  | PN2 |  | ZINC94217163 | 147 | 87 | -36.2132 | -7.15 | 1.590 | 1.587 | 1.589 | 1.570 | **1.301** |
|  |  | PN2 |  | ZINC94927184 | 2 | 150 | -43.1395 | -6.92 | 1.910 | 1.947 | **1.869** | 1.870 | 2.021 |
|  |  | PN2 |  | ZINC95022396 | 132 | 42 | -36.3742 | -7.43 | **2.006** | 2.014 | 2.014 | 2.012 | 2.016 |
|  |  |  |  |  |  |  |  |  |  |  |  |  |  |
|  |  | PN2 | 11 | ZINC14060343 | 38 | 128 | -37.0963 | -6.99 | 1.446 | **1.427** | 1.443 | 1.435 | 1.471 |
|  |  | PN2 |  | ZINC35935889 | 135 | 2 | -35.5389 | -8.47 | 2.698 | 2.714 | 2.406 | 2.483 | **1.551** |
|  |  | PN2 |  | ZINC72148214 | 115 | 55 | -35.7789 | -7.29 | 1.650 | 1.686 | 1.697 | 1.621 | **1.498** |
|  |  | PN2 |  | ZINC72264974 | 114 | 46 | -35.7853 | -7.36 | 1.545 | **1.501** | 1.502 | 1.545 | 1.645 |
|  |  | PN2 |  | ZINC94629031 | 103 | 112 | -35.8935 | -7.03 | 2.716 | 1.894 | 1.885 | **1.875** | 1.887 |
|  |  | PN2 |  | ZINC94927184 | 29 | 150 | -37.4455 | -6.92 | **1.542** | 1.596 | 1.543 | 1.618 | 1.730 |
|  |  | PN2 |  | ZINC73641145 | 61 | 28 | -36.6770 | -7.6 | 1.368 | 1.373 | **1.351** | 1.356 | 1.369 |
|  |  | PN2 |  | ZINC72129411 | 32 | 44 | -37.2583 | -7.41 | **1.68** | 1.693 | 1.696 | 1.686 | 1.682 |
|  |  | PN2 |  | ZINC72107957 | 87 | 8 | -36.2157 | -8.16 | **1.322** | 1.401 | 1.418 | 1.538 | 1.529 |
|  |  | PN2 |  | ZINC77262630 | 9 | 49 | -38.7278 | -7.35 | **1.378** | 1.431 | 1.442 | 1.432 | 1.418 |
|  |  | PN2 |  | ZINC94937158 | 90 | 60 | -36.0595 | -7.28 | 1.436 | 1.409 | **1.393** | 1.404 | 1.401 |
|  |  |  |  |  |  |  |  |  |  |  |  |  |  |
|  |  | PN4 | 21 | ZINC16932105 | 14 | 32 | -40.6128 | -9.07 | 0.917 | 0.917 | 0.92 | 0.533 | **0.524** |
|  |  | PN4 |  | ZINC12362922 | 10 | 25 | -41.3660 | -9.15 | 0.785 | 0.769 | **0.139** | 0.147 | 0.142 |
|  |  | PN4 |  | ZINC92722391 | 45 | 82 | -38.7467 | -8.61 | 0.408 | **0.394** | 0.411 | 0.497 | 0.407 |
|  |  | PN4 |  | ZINC00149964 | 57 | 29 | -38.0889 | -9.1 | 0.6 | 0.595 | 0.584 | 0.605 | **0.577** |
|  |  | PN4 |  | ZINC06361369 | 81 | 24 | -37.5147 | -9.18 | 0.837 | 0.724 | 0.781 | **0.718** | 0.842 |
|  |  | PN4 |  | ZINC02819777 | 65 | 5 | -37.9025 | -9.73 | 0.685 | 0.701 | **0.65** | 0.679 | 0.664 |
|  |  | PN4 |  | ZINC04829362 | 21 | 97 | -40.3007 | -8.53 | **0.085** | 0.121 | 0.119 | 0.119 | 0.115 |
|  |  | PN4 |  | ZINC04085190 | 39 | 33 | -38.9491 | -9.01 | **0.467** | 0.492 | 0.48 | 0.476 | 0.481 |
|  |  | PN4 |  | ZINC00814199 | 8 | 64 | -41.4340 | -8.77 | 0.553 | 0.51 | 0.514 | **0.508** | 0.526 |
|  |  | PN4 |  | ZINC92179996 | 24 | 2 | -39.9639 | -9.82 | 0.637 | 0.64 | 0.638 | 0.613 | **0.599** |
|  |  | PN4 |  | ZINC05378687 | 86 | 142 | -37.3851 | -8.36 | 0.291 | 0.338 | 0.327 | 0.299 | **0.243** |
|  |  | PN4 |  | ZINC05603964 | 104 | 14 | -36.9289 | -9.38 | 0.772 | 0.745 | 0.746 | **0.732** | 0.752 |
|  |  | PN4 |  | ZINC08913821 | 27 | 121 | -39.8119 | -8.42 | 0.773 | 0.777 | 0.702 | 0.855 | **0.354** |
|  |  | PN4 |  | ZINC26481080 | 106 | 9 | -36.9002 | -9.49 | 0.488 | 0.470 | 0.491 | 0.486 | **0.487** |
|  |  | PN4 |  | ZINC59209390 | 5 | 122 | -43.6131 | -8.42 | 0.368 | 0.360 | **0.334** | 0.336 | 0.378 |
|  |  | PN4 |  | ZINC67489659 | 108 | 108 | -36.8403 | -8.45 | 0.260 | 0.245 | **0.235** | 0.260 | 0.274 |
|  |  | PN4 |  | ZINC72165678 | 87 | 148 | -37.3739 | -8.34 | 0.187 | **0.184** | 0.201 | 0.205 | 0.195 |
|  |  | PN4 |  | ZINC87440345 | 139 | 102 | -36.3448 | -8.5 | 0.748 | 0.737 | 0.724 | 0.734 | **0.375** |
|  |  | PN4 |  | ZINC92722404 | 22 | 116 | -40.2784 | -8.42 | 0.312 | 0.319 | 0.293 | **0.285** | 0.382 |
|  |  | PN4 |  | ZINC92722539 | 114 | 131 | -36.7189 | -8.4 | 0.762 | 0.507 | **0.491** | 0.531 | 0.603 |
|  |  | PN4 |  | ZINC94725877 | 115 | 50 | -36.7033 | -8.86 | 0.261 | 0.274 | 0.275 | 0.266 | **0.258** |
|  |  |  |  |  |  |  |  |  |  |  |  |  |  |
|  |  | PN5 | 7 | ZINC49587767 | 57 | 81 | -36.7268 | -6.59 | 0.655 | **0.592** | 0.631 | 0.67 | 0.62 |
|  |  | PN5 |  | ZINC04334885 | 21 | 35 | -37.8931 | -6.83 | 0.744 | 0.742 | 0.736 | 0.762 | **0.732** |
|  |  | PN5 |  | ZINC72107957 | 87 | 6 | -36.2157 | -7.65 | 0.664 | 0.66 | 0.588 | **0.506** | 0.524 |
|  |  | PN5 |  | ZINC73641145 | 61 | 52 | -36.6770 | -6.73 | 1.32 | 1.444 | 1.372 | 0.804 | **0.741** |
|  |  | PN5 |  | ZINC35935889 | 135 | 2 | -35.5389 | -8.22 | 0.618 | 0.597 | 0.579 | 0.592 | **0.575** |
|  |  | PN5 |  | ZINC72148214 | 115 | 67 | -35.7789 | -6.66 | 0.670 | 0.682 | 0.676 | **0.615** | 0.733 |
|  |  | PN5 |  | ZINC95388070 | 106 | 145 | -35.8458 | -6.4 | **0.955** | 0.974 | 1.244 | 1.245 | 1.200 |
|  |  |  |  |  |  |  |  |  |  |  |  |  |  |
| Phosphoprotein | 4N5B | PP1 | 16 | ZINC85650631 | 79 | 34 | -34.2603 | -6.71 | 0.168 | **0.164** | 0.233 | 0.232 | 0.167 |
|  |  | PP1 |  | ZINC94927184 | 19 | 85 | -35.8097 | -6.55 | **0.176** | 0.19 | 0.177 | 0.395 | 0.385 |
|  |  | PP1 |  | ZINC95384460 | 83 | 49 | -34.1442 | -6.65 | 0.327 | 0.333 | 0.33 | **0.321** | 0.326 |
|  |  | PP1 |  | ZINC72462705 | 1 | 90 | -39.3896 | -6.54 | 0.195 | 0.167 | 0.13 | 0.178 | **0.121** |
|  |  | PP1 |  | ZINC86098248 | 93 | 65 | -34.0772 | -6.6 | **0.105** | 0.108 | 0.109 | 0.108 | 0.107 |
|  |  | PP1 |  | ZINC67884980 | 36 | 23 | -34.9700 | -6.79 | 0.204 | **0.195** | 0.197 | 0.228 | 0.227 |
|  |  | PP1 |  | ZINC77285117 | 38 | 41 | -34.8839 | -6.69 | **0.144** | 0.174 | 0.172 | 0.169 | 0.148 |
|  |  | PP1 |  | ZINC95022396 | 52 | 89 | -34.6239 | -6.54 | 0.261 | 0.264 | **0.256** | 0.261 | 0.266 |
|  |  | PP1 |  | ZINC13016500 | 105 | 144 | -33.9586 | -6.4 | 0.220 | 0.217 | 0.225 | **0.215** | 0.222 |
|  |  | PP1 |  | ZINC24759441 | 128 | 149 | -33.6575 | -6.38 | 0.134 | **0.133** | 0.134 | **0.133** | **0.133** |
|  |  | PP1 |  | ZINC32565459 | 97 | 138 | -34.0351 | -6.41 | 0.348 | 0.350 | 0.339 | 0.339 | **0.338** |
|  |  | PP1 |  | ZINC65370580 | 70 | 147 | -34.3435 | -6.39 | 0.692 | 0.694 | **0.687** | 0.692 | 0.689 |
|  |  | PP1 |  | ZINC65425676 | 65 | 139 | -34.3686 | -6.41 | 0.389 | 0.393 | 0.403 | **0.379** | 0.365 |
|  |  | PP1 |  | ZINC77379208 | 125 | 16 | -33.6660 | -6.87 | 0.580 | 0.582 | 0.588 | 0.586 | **0.575** |
|  |  | PP1 |  | ZINC89195159 | 44 | 125 | -34.7283 | -6.44 | 0.330 | 0.343 | 0.328 | 0.343 | **0.325** |
|  |  | PP1 |  | ZINC89201433 | 104 | 127 | -33.9733 | -6.44 | 0.210 | 0.207 | 0.201 | **0.189** | 0.209 |
|  |  |  |  |  |  |  |  |  |  |  |  |  |  |
|  |  | PP1 | 11 | ZINC20534353 | 37 | 75 | -34.0514 | -6.57 | 0.31 | 0.305 | 0.301 | 0.303 | **0.282** |
|  |  | PP1 |  | ZINC77379208 | 66 | 16 | -33.3778 | -6.87 | **0.191** | 0.215 | 0.214 | 0.2 | 0.197 |
|  |  | PP1 |  | ZINC94927184 | 20 | 83 | -35.1329 | -6.55 | 0.589 | 0.564 | 0.588 | 0.594 | **0.537** |
|  |  | PP1 |  | ZINC65405061 | 40 | 55 | -33.9598 | -6.62 | **0.236** | 0.239 | 0.239 | 0.24 | 0.242 |
|  |  | PP1 |  | ZINC72462705 | 9 | 89 | -36.1022 | -6.54 | **0.14** | 0.227 | 0.2 | 0.229 | 0.198 |
|  |  | PP1 |  | ZINC77285117 | 12 | 39 | -35.8396 | -6.69 | 0.177 | 0.136 | 0.152 | **0.13** | 0.18 |
|  |  | PP1 |  | ZINC24759441 | 82 | 150 | -33.1239 | -6.38 | 0.144 | 0.144 | 0.144 | 0.144 | **0.143** |
|  |  | PP1 |  | ZINC32565459 | 35 | 138 | -34.2068 | -6.41 | **0.318** | 0.330 | 0.320 | 0.320 | 0.319 |
|  |  | PP1 |  | ZINC72133204 | 133 | 26 | -32.4142 | -6.75 | **0.231** | 0.233 | 0.234 | **0.231** | **0.231** |
|  |  | PP1 |  | ZINC86098246 | 114 | 72 | -32.6182 | -6.58 | 0.234 | **0.185** | 0.208 | 0.226 | 0.210 |
|  |  | PP1 |  | ZINC95448845 | 88 | 149 | -33.0482 | -6.38 | 0.530 | 0.572 | **0.452** | 0.509 | 0.547 |
|  |  |  |  |  |  |  |  |  |  |  |  |  |  |
|  |  | PP1 | 4 | ZINC72462705 | 10 | 89 | -62.9671 | -6.54 | 7.624 | **7.591** | 7.638 | 7.599 | 7.632 |
|  |  | PP1 |  | ZINC94927184 | 7 | 84 | -64.3531 | -6.55 | 7.708 | 7.709 | 7.716 | 7.587 | **7.584** |
|  |  | PP1 |  | ZINC31394118 | 90 | 111 | -56.0937 | -6.48 | 7.653 | 7.661 | 7.661 | **7.649** | **7.649** |
|  |  | PP1 |  | ZINC72438392 | 104 | 71 | -55.1363 | -6.59 | 7.514 | 7.522 | 7.517 | 7.522 | **7.512** |
|  |  |  |  |  |  |  |  |  |  |  |  |  |  |
|  |  | PP2 | 24 | ZINC04722076 | 5 | 30 | -68.4787 | -9.91 | **0.515** | **0.515** | **0.515** | **0.515** | **0.515** |
|  |  | PP2 |  | ZINC71260677 | 70 | 34 | -56.1610 | -9.77 | 0.218 | 0.217 | 0.213 | **0.208** | 0.209 |
|  |  | PP2 |  | ZINC94927184 | 7 | 49 | -64.2946 | -9.54 | **2.032** | 2.055 | 2.414 | 2.317 | 2.423 |
|  |  | PP2 |  | ZINC67895025 | 62 | 92 | -56.8376 | -9.25 | **0.66** | 0.672 | 0.664 | **0.66** | **0.66** |
|  |  | PP2 |  | ZINC19362297 | 74 | 67 | -55.8450 | -9.41 | 0.301 | **0.299** | **0.299** | 0.314 | 0.299 |
|  |  | PP2 |  | ZINC01584645 | 10 | 35 | -62.4865 | -9.76 | **0.943** | 1.031 | 1.027 | 1.014 | 1.073 |
|  |  | PP2 |  | ZINC86094832 | 29 | 73 | -59.6800 | -9.38 | 0.507 | **0.346** | 0.369 | 0.658 | 0.523 |
|  |  | PP2 |  | ZINC86095599 | 34 | 97 | -59.0738 | -9.21 | **0.118** | 0.165 | 0.155 | 0.133 | 0.133 |
|  |  | PP2 |  | ZINC92209154 | 35 | 29 | -59.0716 | -9.92 | 0.632 | 0.638 | **0.62** | 0.748 | 0.787 |
|  |  | PP2 |  | ZINC95221243 | 47 | 33 | -57.9412 | -9.77 | **0.949** | 0.955 | 0.961 | 0.978 | 0.957 |
|  |  | PP2 |  | ZINC91252717 | 2 | 1 | -71.4697 | -14.3 | 0.437 | 0.438 | **0.427** | 0.43 | 0.431 |
|  |  | PP2 |  | ZINC35605802 | 38 | 15 | -58.9016 | -10.29 | 0.114 | 0.115 | **0.111** | 0.116 | 0.116 |
|  |  | PP2 |  | ZINC72143751 | 91 | 27 | -55.1678 | -10.11 | **2.07** | **2.07** | 2.072 | 2.075 | 2.071 |
|  |  | PP2 |  | ZINC72462705 | 14 | 24 | -61.4933 | -10.16 | 0.955 | 0.96 | **0.866** | 0.887 | 0.89 |
|  |  | PP2 |  | ZINC19320365 | 132 | 16 | -53.0723 | -10.25 | 0.304 | 0.304 | **0.303** | **0.303** | 0.305 |
|  |  | PP2 |  | ZINC19328716 | 59 | 105 | -56.9384 | -9.16 | 0.241 | 0.233 | 0.257 | 0.246 | **0.212** |
|  |  | PP2 |  | ZINC36108799 | 122 | 109 | -53.5489 | -9.14 | **0.249** | **0.249** | 0.259 | 0.259 | **0.249** |
|  |  | PP2 |  | ZINC39417829 | 48 | 113 | -57.8970 | -9.13 | 0.422 | **0.368** | 0.496 | 0.501 | 0.498 |
|  |  | PP2 |  | ZINC41206867 | 54 | 129 | -57.1939 | -9.03 | **0.611** | 0.690 | 0.650 | 0.679 | 0.685 |
|  |  | PP2 |  | ZINC86094658 | 21 | 103 | -60.3732 | -9.19 | 0.458 | 0.444 | 0.421 | 0.543 | **0.216** |
|  |  | PP2 |  | ZINC86662794 | 111 | 111 | -53.9779 | -9.14 | 0.239 | **0.200** | 0.202 | 0.234 | 0.236 |
|  |  | PP2 |  | ZINC86680029 | 112 | 91 | -53.9641 | -9.25 | 0.503 | **0.502** | **0.502** | **0.502** | 0.513 |
|  |  | PP2 |  | ZINC86730664 | 18 | 102 | -60.9041 | -9.19 | 0.954 | 0.956 | 0.946 | **0.913** | **0.913** |
|  |  | PP2 |  | ZINC87254662 | 81 | 135 | -55.7055 | -8.99 | 0.594 | 0.593 | **0.592** | 0.599 | 0.606 |
|  |  |  |  |  |  |  |  |  |  |  |  |  |  |
| Fusion protein | 5EVM | PF1 | 3 | ZINC19558876 | 118 | 141 | -42.6413 | -8.03 | 5.815 | **5.791** | 5.835 | 5.841 | 5.828 |
|  |  | PF1 |  | ZINC44831966 | 140 | 23 | -42.3345 | -8.68 | 5.651 | 5.665 | 5.639 | **5.620** | 5.624 |
|  |  | PF1 |  | ZINC63411510 | 15 | 34 | -46.6526 | -8.54 | 59.52 | 59.61 | 59.00 | 58.96 | 59.01 |
|  |  |  |  |  |  |  |  |  |  |  |  |  |  |
|  |  | PF2 | 7 | ZINC94725877 | 91 | 43 | -38.3918 | -8.82 | 0.397 | 0.405 | 0.4 | 0.374 | **0.311** |
|  |  | PF2 |  | ZINC72131030 | 74 | 4 | -38.7232 | -9.52 | **0.45** | 0.452 | 0.473 | 0.471 | 0.457 |
|  |  | PF2 |  | ZINC93518195 | 68 | 92 | -38.8302 | -8.5 | 0.582 | 0.582 | 0.582 | 0.585 | **0.578** |
|  |  | PF2 |  | ZINC65418720 | 7 | 94 | -42.7932 | -8.49 | **0.541** | 0.566 | 0.579 | 0.567 | 0.578 |
|  |  | PF2 |  | ZINC34083754 | 65 | 35 | -38.9075 | -8.9 | 0.362 | 0.369 | 0.385 | 0.357 | **0.343** |
|  |  | PF2 |  | ZINC00467624 | 52 | 127 | -39.1657 | -8.4 | 0.340 | **0.313** | **0.313** | 0.332 | 0.322 |
|  |  | PF2 |  | ZINC04337208 | 141 | 11 | -37.7391 | -9.2 | 0.434 | 0.434 | 0.434 | 0.430 | **0.425** |
|  |  |  |  |  |  |  |  |  |  |  |  |  |  |
| Matrix protein | Monomer of modeled dimer | PM1 | 1 | ZINC02511792 | 41 | 87 | -39.7644 | -7.45 | 0.543 | 0.503 | 0.599 | 0.504 | **0.48** |
|  |  |  |  |  |  |  |  |  |  |  |  |  |  |
|  |  | PM1 | 3 | ZINC02819777 | 26 | 22 | -36.5827 | -7.85 | 1.689 | 1.668 | **1.664** | 1.674 | 1.683 |
|  |  | PM1 |  | ZINC00344036 | 84 | 120 | -32.7182 | -7.34 | 0.709 | **0.708** | 0.710 | 0.710 | 0.710 |
|  |  | PM1 |  | ZINC05603964 | 23 | 129 | -37.1505 | -7.3 | 1.941 | 1.953 | 1.955 | 1.924 | **1.919** |
|  |  |  |  |  |  |  |  |  |  |  |  |  |  |
|  |  | PM2 | 23 | ZINC26481080 | 53 | 25 | -45.3372 | -8.55 | 0.415 | 0.394 | 0.414 | 0.389 | **0.388** |
|  |  | PM2 |  | ZINC12362922 | 78 | 29 | -44.4204 | -8.51 | **0.183** | 0.186 | 0.185 | **0.183** | **0.183** |
|  |  | PM2 |  | ZINC00814199 | 14 | 7 | -49.4513 | -8.97 | 0.624 | 0.621 | 0.622 | **0.619** | 0.624 |
|  |  | PM2 |  | ZINC31165406 | 34 | 20 | -46.4279 | -8.61 | 0.4 | 0.392 | **0.236** | 0.381 | 0.47 |
|  |  | PM2 |  | ZINC00149964 | 31 | 16 | -47.1346 | -8.67 | 0.624 | 0.621 | 0.622 | **0.619** | 0.624 |
|  |  | PM2 |  | ZINC01725633 | 20 | 15 | -48.5007 | -8.68 | 0.409 | 0.42 | 0.204 | 0.399 | **0.147** |
|  |  | PM2 |  | ZINC16932105 | 37 | 34 | -46.1510 | -8.48 | 0.315 | **0.314** | 0.315 | 0.315 | **0.314** |
|  |  | PM2 |  | ZINC71789643 | 73 | 30 | -44.5767 | -8.51 | 0.364 | 0.352 | 0.366 | **0.341** | 0.362 |
|  |  | PM2 |  | ZINC93518353 | 97 | 100 | -43.7746 | -8.02 | 0.41 | 0.411 | **0.397** | 0.464 | 0.492 |
|  |  | PM2 |  | ZINC91497887 | 87 | 67 | -44.0917 | -8.21 | 0.192 | 0.187 | 0.19 | **0.185** | 0.2 |
|  |  | PM2 |  | ZINC02819777 | 90 | 28 | -44.0287 | -8.51 | 0.475 | 0.469 | 0.471 | **0.414** | 0.482 |
|  |  | PM2 |  | ZINC19735365 | 86 | 93 | -44.1054 | -8.04 | **0.529** | **0.529** | 0.532 | 0.531 | 0.532 |
|  |  | PM2 |  | ZINC04085190 | 40 | 113 | -45.9788 | -7.97 | **0.352** | 0.354 | **0.352** | 0.354 | 0.354 |
|  |  | PM2 |  | ZINC04829362 | 79 | 107 | -44.3996 | -7.99 | 0.315 | 0.313 | 0.316 | **0.312** | 0.313 |
|  |  | PM2 |  | ZINC05331903 | 144 | 22 | -42.7511 | -8.58 | **0.463** | 0.474 | 0.469 | 0.470 | 0.496 |
|  |  | PM2 |  | ZINC05372521 | 48 | 139 | -45.6329 | -7.88 | 0.291 | 0.291 | 0.332 | 0.331 | **0.286** |
|  |  | PM2 |  | ZINC05382414 | 29 | 103 | -47.3167 | -8.01 | 0.539 | 0.526 | **0.438** | 0.443 | 0.447 |
|  |  | PM2 |  | ZINC20154773 | 113 | 36 | -43.4471 | -8.44 | 0.171 | 0.165 | 0.165 | **0.164** | 0.174 |
|  |  | PM2 |  | ZINC22130393 | 139 | 30 | -42.8524 | -8.51 | 0.162 | **0.156** | 0.161 | 0.162 | 0.159 |
|  |  | PM2 |  | ZINC45070221 | 52 | 130 | -45.3486 | -7.91 | 0.164 | 0.184 | **0.142** | 0.166 | 0.161 |
|  |  | PM2 |  | ZINC63781317 | 115 | 23 | -43.4275 | -8.57 | 0.601 | 0.212 | 0.215 | 0.212 | 0.123 |
|  |  | PM2 |  | ZINC72131030 | 147 | 45 | -42.6896 | -8.39 | 0.361 | 0.304 | 0.523 | 0.428 | **0.295** |
|  |  | PM2 |  | ZINC92722404 | 74 | 143 | -44.5125 | -7.87 | **0.507** | 0.539 | 0.570 | 0.523 | 0.584 |
|  |  |  |  |  |  |  |  |  |  |  |  |  |  |
|  |  | PM2 | 26 | ZINC26481080 | 20 | 24 | -45.2565 | -8.55 | 0.352 | 0.355 | 0.364 | **0.293** | **0.293** |
|  |  | PM2 |  | ZINC93518353 | 36 | 98 | -43.0793 | -8.02 | 0.389 | 0.387 | 0.385 | 0.447 | **0.35** |
|  |  | PM2 |  | ZINC00814199 | 27 | 7 | -44.3983 | -8.97 | 0.655 | **0.645** | 0.653 | 0.652 | 0.682 |
|  |  | PM2 |  | ZINC00149964 | 12 | 16 | -46.6062 | -8.67 | **0.452** | 0.454 | 0.459 | 0.457 | 0.457 |
|  |  | PM2 |  | ZINC02819777 | 92 | 29 | -40.6191 | -8.51 | 0.315 | 0.316 | 0.337 | 0.384 | **0.302** |
|  |  | PM2 |  | ZINC31165406 | 22 | 20 | -45.0060 | -8.61 | 0.424 | 0.42 | **0.297** | 0.398 | 0.446 |
|  |  | PM2 |  | ZINC02511792 | 68 | 67 | -41.3050 | -8.21 | **0.165** | 0.187 | 0.185 | 0.173 | 0.166 |
|  |  | PM2 |  | ZINC91497887 | 89 | 66 | -40.6680 | -8.21 | **0.247** | **0.247** | 0.254 | 0.253 | 0.252 |
|  |  | PM2 |  | ZINC00129345 | 66 | 95 | -41.3710 | -8.04 | 0.423 | 0.454 | **0.41** | 0.441 | 0.42 |
|  |  | PM2 |  | ZINC01725633 | 58 | 15 | -41.7507 | -8.68 | 0.423 | 0.418 | **0.415** | 0.418 | 0.447 |
|  |  | PM2 |  | ZINC04020772 | 23 | 46 | -44.9580 | -8.37 | 0.457 | 0.461 | 0.452 | **0.449** | 0.461 |
|  |  | PM2 |  | ZINC04085190 | 42 | 113 | -42.8898 | -7.97 | 0.494 | 0.496 | 0.495 | 0.495 | **0.490** |
|  |  | PM2 |  | ZINC04829362 | 64 | 108 | -41.4436 | -7.99 | 0.218 | 0.226 | 0.216 | **0.214** | 0.227 |
|  |  | PM2 |  | ZINC04962728 | 142 | 43 | -39.4863 | -8.4 | 0.550 | 0.552 | 0.550 | 0.551 | **0.549** |
|  |  | PM2 |  | ZINC05382414 | 8 | 103 | -48.0795 | -8.01 | 0.537 | 0.518 | 0.416 | 0.410 | **0.404** |
|  |  | PM2 |  | ZINC12362922 | 148 | 29 | -39.3891 | -8.51 | 0.387 | 0.388 | 0.387 | **0.385** | 0.386 |
|  |  | PM2 |  | ZINC20154773 | 133 | 36 | -39.7094 | -8.44 | 1.956 | 1.950 | **1.946** | **1.945** | 1.964 |
|  |  | PM2 |  | ZINC45070221 | 59 | 131 | -41.6704 | -7.91 | 0.389 | **0.383** | 0.400 | 0.390 | 0.393 |
|  |  | PM2 |  | ZINC45796058 | 94 | 121 | -40.5724 | -7.95 | 0.734 | 0.734 | 0.739 | 0.738 | **0.728** |
|  |  | PM2 |  | ZINC65407126 | 131 | 18 | -39.7992 | -8.62 | 1.666 | 1.666 | **1.635** | 1.642 | 1.644 |
|  |  | PM2 |  | ZINC83236053 | 112 | 118 | -40.1324 | -7.96 | 0.629 | 0.581 | 0.318 | 0.322 | **0.317** |
|  |  | PM2 |  | ZINC87017834 | 130 | 115 | -39.8160 | -7.97 | 0.288 | 0.312 | 0.307 | **0.285** | 0.288 |
|  |  | PM2 |  | ZINC92711149 | 119 | 77 | -40.0153 | -8.13 | 0.376 | 0.377 | **0.375** | 0.376 | **0.375** |
|  |  | PM2 |  | ZINC92722404 | 33 | 144 | -43.8065 | -7.87 | 0.469 | 0.484 | 0.514 | **0.462** | 0.544 |
|  |  | PM2 |  | ZINC94936845 | 32 | 106 | -43.9738 | -7.99 | 0.420 | 0.412 | 0.403 | 0.332 | **0.306** |
|  |  | PM2 |  | ZINC95359457 | 103 | 109 | -40.3436 | -7.99 | 1.756 | **1.744** | 1.745 | 1.777 | 1.749 |
|  |  |  |  |  |  |  |  |  |  |  |  |  |  |
|  |  | PM3 | 10 | ZINC49453727 | 86 | 70 | -38.7917 | -6.56 | 1.949 | **1.94** | 1.946 | 1.969 | 1.992 |
|  |  | PM3 |  | ZINC83328368 | 25 | 33 | -40.6870 | -6.82 | 1.775 | 1.781 | 1.775 | **1.74** | 1.743 |
|  |  | PM3 |  | ZINC72131030 | 72 | 19 | -38.9782 | -6.9 | 1.892 | **1.89** | 1.914 | 1.893 | 1.905 |
|  |  | PM3 |  | ZINC20390482 | 60 | 94 | -39.1807 | -6.48 | 1.821 | 1.821 | 1.821 | **1.819** | 1.82 |
|  |  | PM3 |  | ZINC63781317 | 51 | 53 | -39.3671 | -6.66 | 1.839 | 1.829 | 1.829 | **1.746** | 1.796 |
|  |  | PM3 |  | ZINC20163996 | 12 | 72 | -42.2337 | -6.56 | 1.896 | 1.896 | 1.894 | **1.893** | **1.893** |
|  |  | PM3 |  | ZINC00189011 | 112 | 142 | -38.2955 | -6.38 | **1.644** | 1.648 | 1.649 | 1.650 | 1.653 |
|  |  | PM3 |  | ZINC86864968 | 134 | 10 | -38.0403 | -7.23 | 1.744 | 1.705 | **1.680** | 1.723 | 1.780 |
|  |  | PM3 |  | ZINC89949696 | 55 | 128 | -39.2591 | -6.4 | 1.941 | 1.944 | 1.944 | 1.494 | **1.479** |
|  |  | PM3 |  | ZINC95008629 | 93 | 122 | -38.6525 | -6.41 | 1.973 | 1.977 | **1.972** | 1.978 | 1.975 |
|  |  |  |  |  |  |  |  |  |  |  |  |  |  |
|  |  | PM3 | 8 | ZINC20163996 | 70 | 69 | -33.0270 | -6.56 | 2.471 | 2.47 | 2.466 | 2.464 | **2.463** |
|  |  | PM3 |  | ZINC05603964 | 23 | 27 | -37.1505 | -6.85 | 3.517 | 3.53 | 3.526 | 3.524 | **3.515** |
|  |  | PM3 |  | ZINC72131030 | 48 | 19 | -34.1461 | -6.9 | 1.832 | 1.837 | **1.793** | 1.827 | 1.803 |
|  |  | PM3 |  | ZINC91497887 | 9 | 43 | -39.8472 | -6.73 | 3.406 | 3.389 | 3.426 | **3.384** | 3.425 |
|  |  | PM3 |  | ZINC73736970 | 88 | 24 | -32.4629 | -6.86 | 3.397 | **3.339** | 3.412 | 3.433 | 3.427 |
|  |  | PM3 |  | ZINC02819777 | 26 | 15 | -36.5827 | -7.1 | 3.317 | 3.338 | **3.303** | 3.342 | 3.356 |
|  |  | PM3 |  | ZINC01418749 | 113 | 133 | -31.9688 | -6.39 | 1.652 | 1.644 | **1.641** | 1.654 | 1.649 |
|  |  | PM3 |  | ZINC33295102 | 21 | 121 | -37.3533 | -6.41 | 3.370 | 3.367 | **3.365** | 3.384 | 3.384 |
