# Supplementary material for: Predicting and designing therapeutics against the Nipah virus
Source: PLoS Negl Trop Dis. 2019 Dec 12;13(12):e0007419. doi: 10.1371/journal.pntd.0007419 (PMC6907750; doi:10.1371/journal.pntd.0007419)
Supplement: S10 Table — Number of common hydrogen bonds indicates the number of hydrogen bonds that are common between the predicted poses of the ligand from Autodock4 and DOCK6.8. (DOCX) [file pntd.0007419.s010.docx]

| **Sr no** | **Protein Name** | **Pocket Number** | **ZINC ID** | **Number of Hydrogen bonds for DOCK6.8** | **Number of Hydrogen bonds for Autodock4** | **Number of common Hydrogen bonds** |
| --- | --- | --- | --- | --- | --- | --- |
| 1 | Nucleoprotein | PN21 | ZINC94258558 | 4 | 3 | 3 |
| 2 | Nucleoprotein | PN21 | ZINC73641145 | 7 | 7 | 4 |
| 3 | Nucleoprotein | PN4 | ZINC12362922 | 5 | 7 | 2 |
| 4 | Phosphoprotein | PP11 | ZINC72462705 | 1 | 2 | 1 |
| 5 | Phosphoprotein | PP11 | ZINC86098248 | 1 | 1 | 0 |
| 6 | Phosphoprotein | PP11 | ZINC77285117 | 1 | 2 | 1 |
| 7 | Phosphoprotein | PP12 | ZINC72462705 | 1 | 2 | 0 |
| 8 | Phosphoprotein | PP12 | ZINC77285117 | 0 | 1 | 0 |
| 9 | Phosphoprotein | PP2 | ZINC86095599 | 1 | 1 | 0 |
| 10** | Phosphoprotein | PP2 | ZINC91252717 | 3 | 2 | 0 |
| 11 | Phosphoprotein | PP2 | ZINC35605802 | 2 | 4 | 2 |
| 12 | Nucleoprotein | P12 | ZINC16545537 | 2 | 4 | 2 |
| 13 | Nucleoprotein | P12 | ZINC63959595 | 4 | 4 | 3 |
| 14 | Nucleoprotein | PN21 | ZINC91932783 | 3 | 5 | 3 |
| 15 | Nucleoprotein | PN4 | ZINC12362922 | 5 | 7 | 2 |
| 16 | Nucleoprotein | PN4 | ZINC04829362 | 5 | 6 | 5 |
| 17 | Phosphoprotein | PP11 | ZINC24759441 | 0 | 1 | 0 |
| 18 | Phosphoprotein | PP11 | ZINC77285117 | 0 | 2 | 0 |
| 19 | Phosphoprotein | PP12 | ZINC24759441 | 0 | 1 | 0 |
| 20 | Phosphoprotein | PP21 | ZINC86095599 | 1 | 1 | 0 |
| 22 | Matrix protein | PM21 | ZINC45070221 | 3 | 3 | 2 |
| 23 | Matrix protein | PM21 | ZINC01725633 | 4 | 5 | 2 |

** The RMSD between DOCK and Autodock is 0.427 nm (greater than the cutoff). This entry is included as the rank for this ligand DOCK is 2 and Autodock is 1, indicating higher confidence in the prediction
