# Supplementary material for: Predicting and designing therapeutics against the Nipah virus
Source: PLoS Negl Trop Dis. 2019 Dec 12;13(12):e0007419. doi: 10.1371/journal.pntd.0007419 (PMC6907750; doi:10.1371/journal.pntd.0007419)
Supplement: S11 Table — The binding pocket has been mentioned in parenthesis. (DOCX) [file pntd.0007419.s011.docx]

| **Sr. No.** | **ZINC ID** | **Pocket Number** |
| --- | --- | --- |
| 1 | ZINC72148214 | PN23,PN5 |
| 2 | ZINC05603964 | PN4,PM12,PM32 |
| 3 | ZINC93518353 | PM21,PM22 |
| 4 | ZINC04829362 | PN4,PM21,PM22 |
| 5 | ZINC77285117 | PP11,PP11,PP12 |
| 6 | ZINC92722404 | PN4,PM21,PM22 |
| 7 | ZINC16932105 | PN4,PM21 |
| 8 | ZINC34083937 | PN11,PN12 |
| 9 | ZINC12362922 | PN4,PM21,PM22 |
| 10 | ZINC00814199 | PN4,PM21,PM22 |
| 11 | ZINC72462705 | PP11,PP12,PP13,PP2 |
| 12 | ZINC95022396 | PN21,PN22,PP11 |
| 13 | ZINC94927184 | PN21,PN22,PN23,PP11,PP12,PP13,PP2 |
| 14 | ZINC72131030 | PF2,PM21,PM31,PM32 |
| 15 | ZINC20163996 | PM31,PM32 |
| 16 | ZINC32565459 | PP11,PP12 |
| 17 | ZINC02511792 | PN12,PM11,PM22 |
| 18 | ZINC94725877 | PN4,PF2 |
| 19 | ZINC63781317 | PM21,PM31 |
| 20 | ZINC94217163 | PG2,PN22 |
| 21 | ZINC24759441 | PP11,PP12 |
| 22 | ZINC91932783 | PN21,PN22 |
| 23 | ZINC20154773 | PM21,PM22 |
| 24 | ZINC91497887 | PM21,PM22,PM32 |
| 25 | ZINC77262630 | PN21,PN22 |
| 26 | ZINC45070221 | PM21,PM22 |
| 27 | ZINC31165406 | PM21,PM22 |
| 28 | ZINC26481080 | PN4,PM21,PM22 |
| 29 | ZINC04580552 | PG2,PN21 |
| 30 | ZINC72264974 | PG1,PN21,PN23 |
| 31 | ZINC63411510 | PG1,PF1 |
| 32 | ZINC05382414 | PN12,PM21,PM22 |
| 33 | ZINC02819777 | PN4,PM12,PM21,PM22,PM32 |
| 34 | ZINC72133204 | PN22,PP12 |
| 35 | ZINC72129411 | PN22,PN22 |
| 36 | ZINC35935889 | PN23,PN5 |
| 37 | ZINC72107957 | PN21,PN22,PN5 |
| 38 | ZINC77379208 | PP11,PP12 |
| 39 | ZINC92722391 | PN12,PN4 |
| 40 | ZINC94937158 | PN21,PN22 |
| 41 | ZINC92722539 | PN12,PN4 |
| 42 | ZINC73641145 | PN21,PN22,PN5 |
| 43 | ZINC04085190 | PN4,PM21,PM22 |
| 44 | ZINC00149964 | PN4,PM21,PM22 |
| 45 | ZINC01725633 | PM21,PM22 |
