# Supplementary material for: Predicting and designing therapeutics against the Nipah virus
Source: PLoS Negl Trop Dis. 2019 Dec 12;13(12):e0007419. doi: 10.1371/journal.pntd.0007419 (PMC6907750; doi:10.1371/journal.pntd.0007419)
Supplement: S12 Table — The binding free energies were not calculated (depicted by -) when the ligand left the binding site in at least 2 out of 3 replicates. CHARMM was only used to run molecular dynamics simulations when the ligand left the binding pocket in AMBER simulations. (DOCX) [file pntd.0007419.s012.docx]

| ZINC ID | Protein | Replicate | Binding free energy as predicted during | |
| --- | --- | --- | --- | --- |
|  |  |  | AMBER simulation (kJ/mol) | CHARMM simulation(kJ/mol) |
| ZINC94258558 | N | 1 | - | -114+/-10 |
|  |  | 2 | - | -86+/-4 |
|  |  | 3 | - | - |
| ZINC73641145 | N | 1 | - | - |
|  |  | 2 | - | - |
|  |  | 3 | - | - |
| ZINC12362922 | N | 1 | -96+/-8 |  |
|  |  | 2 | -100+/-10 |  |
|  |  | 3 | -69+/-6 |  |
| ZINC04829362 | N | 1 | -37+/-7 |  |
|  |  | 2 | 86+/-7 |  |
|  |  | 3 | -101+/-8 |  |
| ZINC72462705 | P | 1 | - | 106+/-4 |
|  |  | 2 | - | 86+/-4 |
|  |  | 3 | - | 98+/-5 |
| ZINC86098248 | P | 1 | 39+/-5 |  |
|  |  | 2 | -65+/-7 |  |
|  |  | 3 | 37+/-3 |  |
| ZINC77285117 | P | 1 | - | - |
|  |  | 2 | - | - |
|  |  | 3 | - | - |
| ZINC86095599 | P | 1 | -196+/-7 |  |
|  |  | 2 | -149+/-10 |  |
|  |  | 3 | -153+/-14 |  |
| ZINC35605802 | P | 1 | -14+/-0.5 |  |
|  |  | 2 | 1+/-1 |  |
|  |  | 3 | -98+/-8 |  |
| ZINC01725633 | M | 1 | - | - |
|  |  | 2 | - | - |
|  |  | 3 | - | - |
