# Supplementary material for: Predicting and designing therapeutics against the Nipah virus
Source: PLoS Negl Trop Dis. 2019 Dec 12;13(12):e0007419. doi: 10.1371/journal.pntd.0007419 (PMC6907750; doi:10.1371/journal.pntd.0007419)
Supplement: S13 Table — The binding free energies were not calculated (depicted by -) when the ligand left the binding site in at least 2 out of 3 replicates. (DOCX) [file pntd.0007419.s013.docx]

| ZINC ID | Protein | Replicate | Binding free energy as predicted from (kJ/mol) | |
| --- | --- | --- | --- | --- |
|  |  |  | DOCK pose (kJ/mol) | Autodock pose (kJ/mol) |
| ZINC00814199 | M | 1 | -153+/-6 | -119+/-8 |
|  |  | 2 | - | -184+/-3 |
|  |  | 3 | -203+/-6 | - |
| ZINC63411510 | G | 1 | - | -44+/-4 |
|  |  | 2 | - | -79+/-4 |
|  |  | 3 | - | -59+/-4 |
| ZINC91252717 | P | 1 | -158+/-9 | -187+/-8 |
|  |  | 2 | -256+/-10 | -196+/-8 |
|  |  | 3 | -251+/-7 | - |
