# Supplementary material for: Predicting and designing therapeutics against the Nipah virus
Source: PLoS Negl Trop Dis. 2019 Dec 12;13(12):e0007419. doi: 10.1371/journal.pntd.0007419 (PMC6907750; doi:10.1371/journal.pntd.0007419)
Supplement: S14 Table — The mutations are mentioned by the residue number followed by the amino acids present in different strains. (DOCX) [file pntd.0007419.s014.docx]

| **Protein** | **Conservation (%)** | **Mutations** |
| --- | --- | --- |
| C | 94.0 | 25 A/V, 33 I/T, 34 K/E, 37 H/P, 39 K/R, 40 I/T, 62 M/V, 67 A/T, 83 K/R, 98 Y/H |
| F | 98.0 | 2 A/V, 6 D/N, 9 Y/C, 11 S/C, 15 I/L, 19 I/M, 42 I/V, 207 S/L, 250 I/T, 252 D/G, 273 S/G |
| G | 94.5 | 3 A/T, 5 S/N, 14 A/T, 20 I/N, 82 M/V, 89 S/G, 172 K/R, 236 K/R, 272 A/T, 274 P/S, 288 S/N, 299 T/V, 304 I/V, 325 S/N, 328 E/G, 329 S/G, 335 L/F, 339 S/N, 344 K/R/M, 384 I/V, 385 A/T, 386 K/E, 421 E/G, 424 P/S, 426 I/V, 427 I/V, 470 Q/L, 481 D/N, 498 K/T, 502 I/V, 545 I/V |
| L | 98.0 | 36 K/R, 71 D/N, 77 I/V, 94 I/T, 112 K/R, 223 T/N, 252 I/V, 533 E/D, 621 K/R, 625 Y/C, 632 S/N, 639 D/N, 640 P/S, 642 Y/N, 658 Y/H, 665 I/T, 703 K/R, 783 K/E, 890 I/V, 1154 I/L, 1157 K/R, 1181 K/R, 1262 K/R, 1494 A/V, 1551 A/S, 1577 I/V, 1645 Y/S/F, 1658 S/N, 1707 M/V, 1748 I/V, 1753 M/V, 1791 A/S, 1801 K/R, 1896 A/T, 2001 M/V, 2027 I/V, 2031 K/R, 2037 D/N, 2039 H/N, 2064 E/D, 2071 Q/H, 2159 C/R, 2216 S/N |
| M | 98.6 | 13 I/M, 26 H/N, 127 I/V, 147 S/G, 331 I/V |
| N | 97.4 | 139 S/R, 188 E/D, 211 Q/R, 345 I/M, 387 D/N, 429 I/V, 432 E/G, 457 D/N, 503 S/N, 505 K/R, 506 D/T, 508 R/G, 520 P/S, 521 A/T |
| P | 89.3 | 41 Q/R, 64 P/S, 69 D/G, 74 S/N, 105 I/T, 139 Y/H, 140 S/T, 147 D/N, 156 M/V, 179 D/N, 183 A/T, 191 I/V, 195 P/L, 196 K/R, 200 D/V, 218 K/R, 219 E/G, 223 D/G, 225 Q/E, 227 S/N, 228 K/R, 269 E/D, 274 S/R, 275 A/V, 276 S/G, 277 R/G, 280 I/N, 283 A/I/V, 285 H/R, 286 I/T, 287 I/L, 292 I/T, 295 S/N, 297 Q/K, 298 I/A, 300 D/G, 303 P/S, 304 A/T, 306 A/V, 310 R/G, 311 P/L, 319 K/E, 320 P/S, 343 Q/R, 351 L/F, 354 S/C, 363 P/L, 365 Y/H, 366 R/W, 367 S/G, 370 R/G, 372 I/R, 377 A/T, 378 K/E, 380 T/V, 381 S/N, 382 D/G, 386 T/N, 388 D/N, 389 K/R, 410 A/E, 421 P/L, 425 S/N, 449 Q/R, 452 A/V, 453 P/S, 455 A/V, 464 A/V, 467 A/V, 590 S/N, 602 I/V, 629 A/T, 635 E/G, 664 I/V, 683 D/G, 687 K/R |
| V | 78.9 | 41 Q/R, 64 P/S, 69 D/G, 74 S/N, 105 I/T, 139 Y/H, 140 S/T, 147 D/N, 156 M/V, 179 D/N, 183 A/T, 191 I/V, 195 P/L, 196 K/R, 200 D/V, 218 K/R, 219 E/G, 223 D/G, 225 Q/E, 227 S/N, 228 K/R, 269 E/D, 275 A/V, 277 R/G, 280 I/N, 283 A/I/V, 285 H/R, 286 I/T, 287 I/L, 292 I/T, 295 S/N, 297 Q/K, 298 I/A, 300 D/G, 303 P/S, 304 A/T, 306 A/V, 311 P/L, 319 K/E, 320 P/S, 343 Q/R, 351 L/F, 354 S/C, 363 P/L, 365 Y/H, 366 R/W, 367 S/G, 370 R/G, 372 I/R, 377 A/T, 380 T/V, 381 S/N, 382 D/G, 386 T/N, 388 D/N, 389 K/R, 408 H/T, 409 R/D, 410 R/E, 411 K/E, 412 Y/I, 413 P/S, 414 I/S, 415 A/C, 416 W/G, 417 T/D, 418 E/G, 419 K/N, 424 P/E/L, 425 E/G, 426 S/W, 427 K/C, 428 S/N, 429 P/G, 431 C/T, 432 S/R, 433 H/R, 434 I/V, 435 R/T, 436 P/G, 437 S/L, 439 P/R, 440 Y/R, 443 C/G, 444 Q/K, 445 S/C, 446 G/V, 447 E/N, 448 A/C, 452 Q/R/, 458 E/V, 459 C/V, 460 K/F, 461 H/E, 462 C/T, 463 D/G |
| W | 79.3 | 41 Q/R, 64 P/S, 69 D/G, 74 S/N, 105 I/T, 139 Y/H, 140 S/T, 147 D/N, 156 M/V, 179 D/N, 183 A/T, 191 I/V, 195 P/L, 196 K/R, 200 D/V, 218 K/R, 219 E/G, 223 D/G, 225 Q/E, 227 S/N, 228 K/R, 269 E/D, 275 A/V, 277 R/G, 280 I/N, 283 A/I/V, 285 H/R, 286 I/T, 287 I/L, 292 I/T, 295 S/N, 297 Q/K, 298 I/A, 300 D/G, 303 P/S, 304 A/T, 306 A/V, 311 P/L, 319 K/E, 320 P/S, 343 Q/R, 351 L/F, 354 S/C, 363 P/L, 365 Y/H, 366 R/W, 367 S/G, 370 R/G, 372 I/R, 377 A/T, 380 T/V, 381 S/N, 382 D/G, 386 T/N, 388 D/N, 389 K/R, 408 A/T, 409 Q/D, 410 E/T, 411 K/R, 412 Y/N, 413 I/P, 414 H/S, 416 A/L, 418 R/T, 419 K/E, 420 T/N, 421 C/V, 422 P/L, 424 S/R, 425 K/R, 426 S/V/N, 427 G/V, 428 A/Q, 429 P/T, 430 R/G, 431 H/M, 432 F/V, 433 R/E, 434 D/G, 435 H/S, 438 Y/T, 439 Q/K, 440 K/E, 441 A/G, 442 K/R 445 S/N, 446 A/M, 447 R/E, 448 R/N, 449 M/V, 450 Q/S/R, 451 L/N |
