# Supplementary material for: Predicting and designing therapeutics against the Nipah virus
Source: PLoS Negl Trop Dis. 2019 Dec 12;13(12):e0007419. doi: 10.1371/journal.pntd.0007419 (PMC6907750; doi:10.1371/journal.pntd.0007419)
Supplement: S15 Table — (DOCX) [file pntd.0007419.s015.docx]

| Peptide inhibitor /ZINC ID | Protein | Docking technique | Force field | Replicate | No of Water molecule | Counter ion | No of counter ion |
| --- | --- | --- | --- | --- | --- | --- | --- |
| IKKSKSYISKAQELL | F |  | AMBER | 1 | 12389 | Cl | 9 |
|  |  |  |  | 2 | 12389 | Cl | 9 |
|  |  |  |  | 3 | 12389 | Cl | 9 |
| RRTAGSTIN | M |  | AMBER | 1 | 39661 | Cl | 14 |
|  |  |  |  | 2 | 39661 | Cl | 14 |
|  |  |  |  | 3 | 39661 | Cl | 14 |
| FSPNLW | G |  | AMBER | 1 | 28107 | Cl | 3 |
|  |  |  |  | 2 | 28107 | Cl | 3 |
| LAPHPSQ | G |  |  | 3 | 28107 | Cl | 3 |
|  |  |  | AMBER | 1 | 27676 | Cl | 2 |
|  |  |  |  | 2 | 27676 | Cl | 2 |
|  |  |  |  | 3 | 27676 | Cl | 2 |
| ZINC63411510 | G | DOCK | AMBER | 1 | 27751 | Cl | 5 |
|  |  |  |  | 2 | 27751 | Cl | 5 |
|  |  |  |  | 3 | 27751 | Cl | 5 |
|  |  | Autodock | AMBER | 1 | 18155 | Cl | 3 |
|  |  |  |  | 2 | 18155 | Cl | 3 |
|  |  |  |  | 3 | 18155 | Cl | 3 |
| ZINC00814199 | M | DOCK | AMBER | 1 | 39673 | Cl | 10 |
|  |  |  |  | 2 | 21328 | Cl | 10 |
|  |  |  |  | 3 | 21328 | Cl | 10 |
|  |  | Autodock | AMBER | 1 | 21329 | Cl | 12 |
|  |  |  |  | 2 | 21329 | Cl | 12 |
|  |  |  |  | 3 | 21329 | Cl | 12 |
| ZINC91252717 | P | DOCK | AMBER | 1 | 78125 | Na | 31 |
|  |  |  |  | 2 | 78125 | Na | 31 |
|  |  |  |  | 3 | 78125 | Na | 31 |
|  |  | Autodock | AMBER | 1 | 26085 | Na | 31 |
|  |  |  |  | 2 | 26085 | Na | 31 |
|  |  |  |  | 3 | 26085 | Na | 31 |
| ZINC94258558 | N | DOCK | AMBER | 1 | 63801 | Cl | 5 |
|  |  |  |  | 2 | 63801 | Cl | 5 |
|  |  |  |  | 3 | 63801 | Cl | 5 |
|  |  | DOCK | CHARMM | 1 | 22447 | Cl | 5 |
|  |  |  |  | 2 | 22447 | Cl | 5 |
|  |  |  |  | 3 | 22447 | Cl | 5 |
| ZINC73641145 | N | DOCK | AMBER | 1 | 21071 | Cl | 5 |
|  |  |  |  | 2 | 21071 | Cl | 5 |
|  |  |  |  | 3 | 21071 | Cl | 5 |
|  |  | DOCK | CHARMM | 1 | 22454 | Cl | 5 |
|  |  |  |  | 2 | 22454 | Cl | 5 |
|  |  |  |  | 3 | 22454 | Cl | 5 |
| ZINC12362922 | N | DOCK | AMBER | 1 | 52005 | Cl | 2 |
|  |  |  |  | 2 | 52005 | Cl | 2 |
|  |  |  |  | 3 | 52005 | Cl | 2 |
| ZINC04829362 | N | DOCK | AMBER | 1 | 52007 | Cl | 2 |
|  |  |  |  | 2 | 21080 | Cl | 2 |
|  |  |  |  | 3 | 21080 | Cl | 2 |
| ZINC72462705 | P | DOCK | AMBER | 1 | 78131 | Na | 32 |
|  |  |  |  | 2 | 78131 | Na | 32 |
|  |  |  |  | 3 | 78131 | Na | 32 |
|  |  |  | CHARMM | 1 | 26099 | Na | 32 |
|  |  |  |  | 2 | 26099 | Na | 32 |
|  |  |  |  | 3 | 26099 | Na | 32 |
| ZINC86098248 | P | DOCK | AMBER | 1 | 78125 | Na | 33 |
|  |  |  |  | 2 | 26105 | Na | 33 |
|  |  |  |  | 3 | 26105 | Na | 33 |
| ZINC77285117 | P | DOCK | AMBER | 1 | 78130 | Na | 33 |
|  |  |  |  | 2 | 26094 | Na | 33 |
|  |  |  |  | 3 | 26094 | Na | 33 |
|  |  |  | CHARMM | 1 | 26090 | Na | 33 |
|  |  |  |  | 2 | 26090 | Na | 33 |
|  |  |  |  | 3 | 26090 | Na | 33 |
| ZINC86095599 | P | DOCK | AMBER | 1 | 26090 | Na | 32 |
|  |  |  |  | 2 | 26090 | Na | 32 |
|  |  |  |  | 3 | 26090 | Na | 32 |
| ZINC35605802 | P | DOCK | AMBER | 1 | 25806 | Na | 32 |
|  |  |  |  | 2 | 25806 | Na | 32 |
|  |  |  |  | 3 | 26088 | Na | 32 |
| ZINC01725633 | M | DOCK | AMBER | 1 | 39673 | Cl | 10 |
|  |  |  |  | 2 | 21328 | Cl | 10 |
|  |  |  |  | 3 | 21328 | Cl | 10 |
|  |  |  | CHARMM | 1 | 21328 | Cl | 10 |
|  |  |  |  | 2 | 21328 | Cl | 10 |
|  |  |  |  | 3 | 21328 | Cl | 10 |
