# Supplementary figures and images for: Predicting and designing therapeutics against the Nipah virus
Source: PLoS Negl Trop Dis. 2019 Dec 12;13(12):e0007419. doi: 10.1371/journal.pntd.0007419 (PMC6907750; doi:10.1371/journal.pntd.0007419)

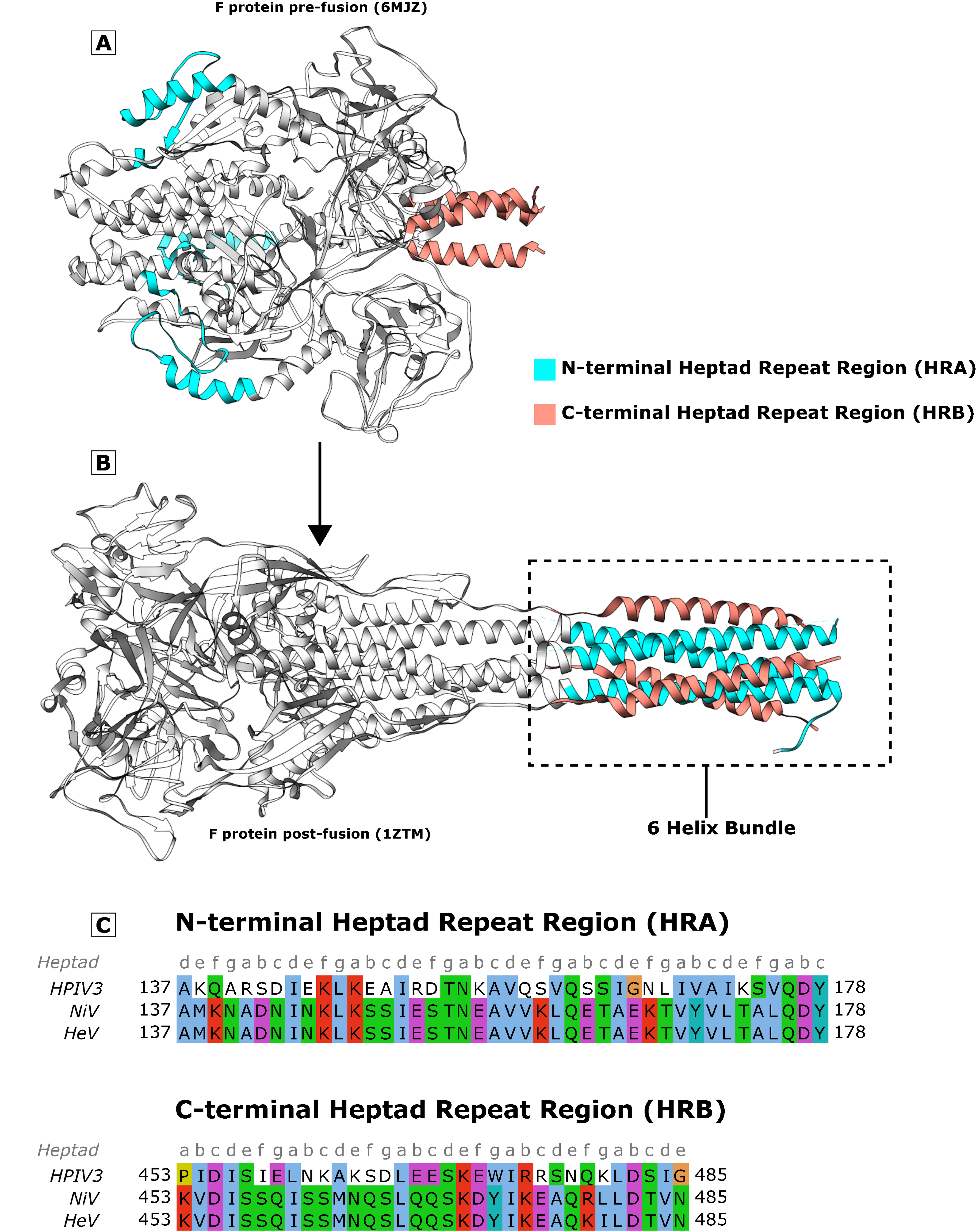

Supplement: S1 Fig — The fusion protein undergoes a large conformational change from the pre-fusion state (A, PDB id: 6MJZ) to post-fusion state (B, PDB id: 1ZTM) to form the 6 helix bundle by interactions between the HRA domain (Salmon ribbon) and HRB domain (Cyan ribbon) heptad repeat regions. (C) Alignment of the heptad repeat regions between fusion protein sequences of the three viruses (Uniprot ids—HPIV3: P06828, NiV: Q9IH63, HeV: O89342). The alignment is color coded based on ClustalX. (TIF) [file pntd.0007419.s016.tif]

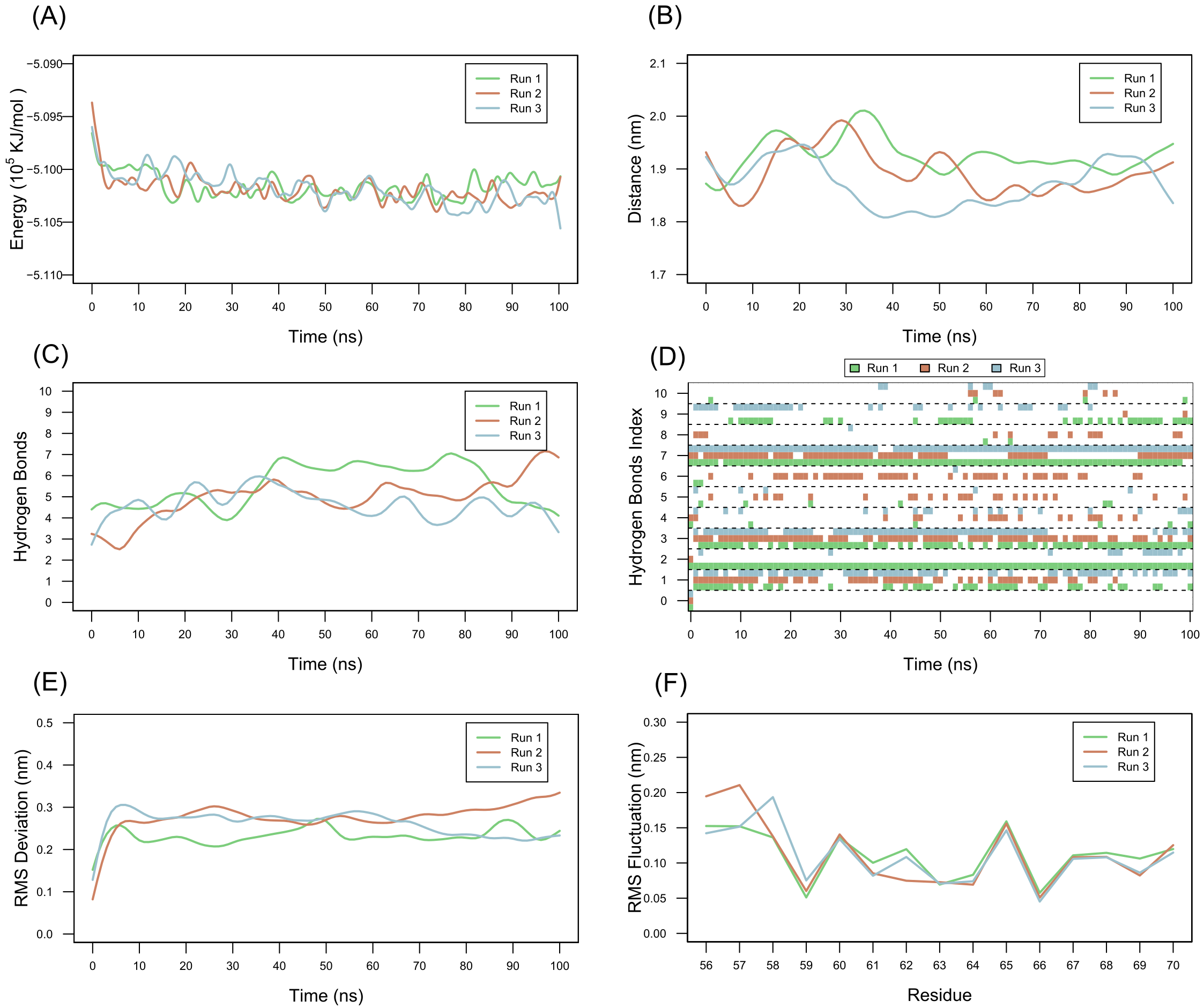

Supplement: S2 Fig — A) Energy of the F protein-inhibitor complex during 100 ns of MD simulation B) Distance of the center of the inhibitor from the center of the F protein during the simulation C) Number of hydrogen bonds between the F protein-inhibitor complex during the simulations D) Plot showing the formation of hydrogen bonds between inhibitor and F protein over 100 ns trajectories. Y axis shows the 11 different hydrogen bonds identified as numbered index (S3 Table). X axis labels time instant during simulation. Each rectangular color box represents the presence of hydrogen bond for a particular run. E) Root mean square deviation (RMSD) # of the designed inhibitor during the simulations F) Root mean square fluctuation (RMSF) # of the inhibitory peptide during the simulations. Each of the simulations were run in triplicate, with each run being color coded as red, green and blue. (# RMSD and RMSF were calculated for the inhibitor by superimposing the protein molecule) (TIF) [file pntd.0007419.s017.tif]

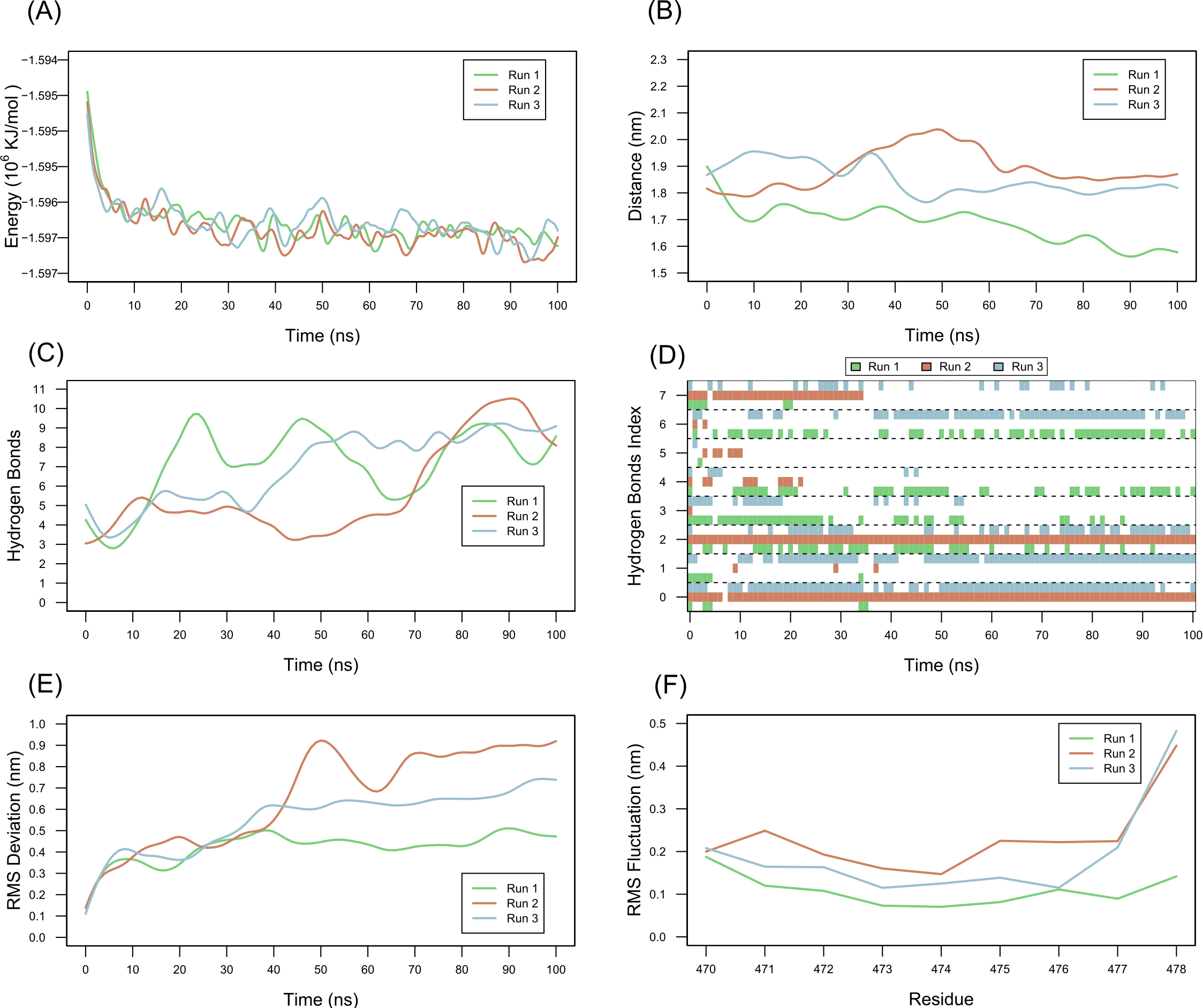

Supplement: S3 Fig — A) Energy of the M protein-inhibitor complex during 100 ns of MD simulation B) Distance of the center of the inhibitor from the center of the M protein during the simulation C) Number of hydrogen bonds between the M protein-inhibitor complex during the simulations D) Plot showing the formation of hydrogen bonds between inhibitor and M protein over 100 ns trajectories. Y axis shows the 8 different hydrogen bonds identified as numbered index (S5 Table). X axis labels time instant during simulation. Each rectangular color box represents the presence of hydrogen bond for a particular run. E) RMSD # of the designed inhibitor during the simulations F) RMSF # of the inhibitory peptide during the simulations. Each of the simulations were run in triplicate, with each run being color coded as red, green and blue. (# RMSD and RMSF were calculated for the inhibitor by superimposing the protein molecule) (TIF) [file pntd.0007419.s018.tif]

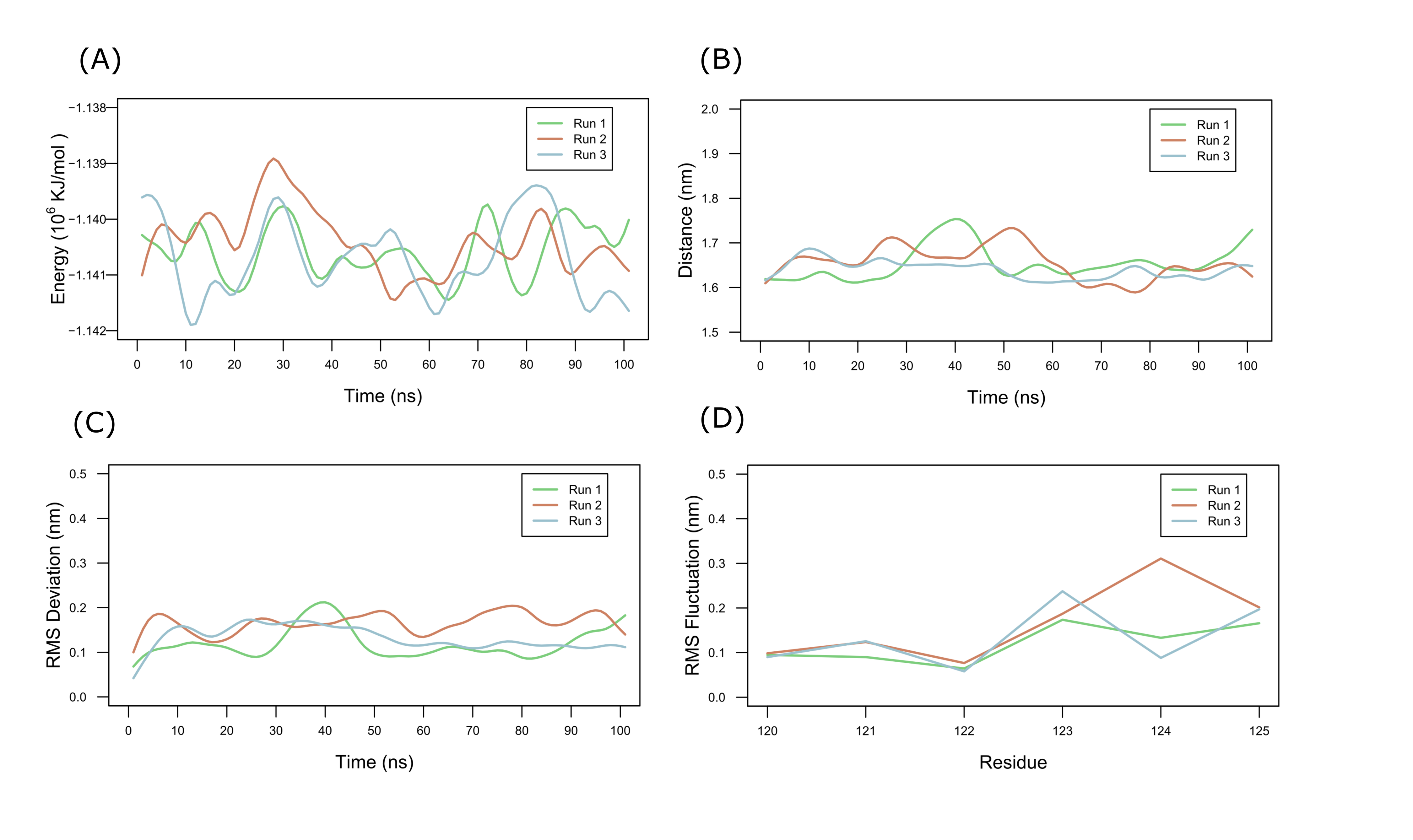

Supplement: S4 Fig — A) Energy of the G protein-FSPNLW inhibitor complex during 100 ns of MD simulation B) Distance of the center of the inhibitor from the center of the G protein during the simulation C) RMSD # of the designed inhibitor during the simulation D) RMSF # of the inhibitory peptide during the simulation. Each of the simulation were run in triplicate, each run being color coded as red, green and blue. (# RMSD and RMSF were calculated for the inhibitor by superimposing the protein molecule) (TIF) [file pntd.0007419.s019.tif]

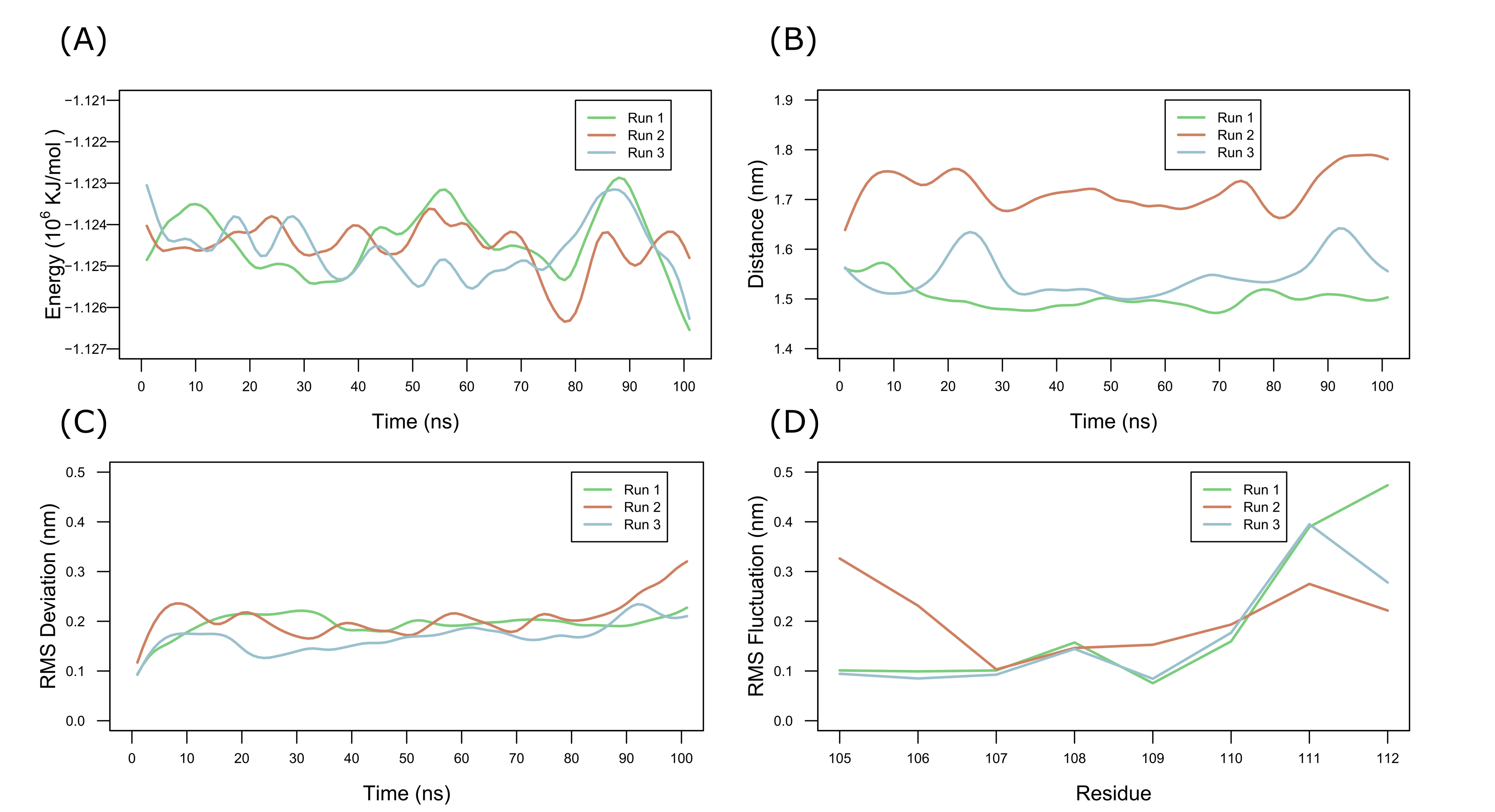

Supplement: S5 Fig — A) Energy of the G protein-LAPHPSQ inhibitor complex during 100 ns of MD simulation B) Distance of the center of the inhibitor from the center of the G protein during the simulation C) RMSD # of the designed inhibitor during the simulation D) RMSF # of the inhibitory peptide during the simulation. Each of the simulation were run in triplicate, each run being color coded as red, green and blue. (# RMSD and RMSF were calculated for the inhibitor by superimposing the protein molecule) (TIF) [file pntd.0007419.s020.tif]

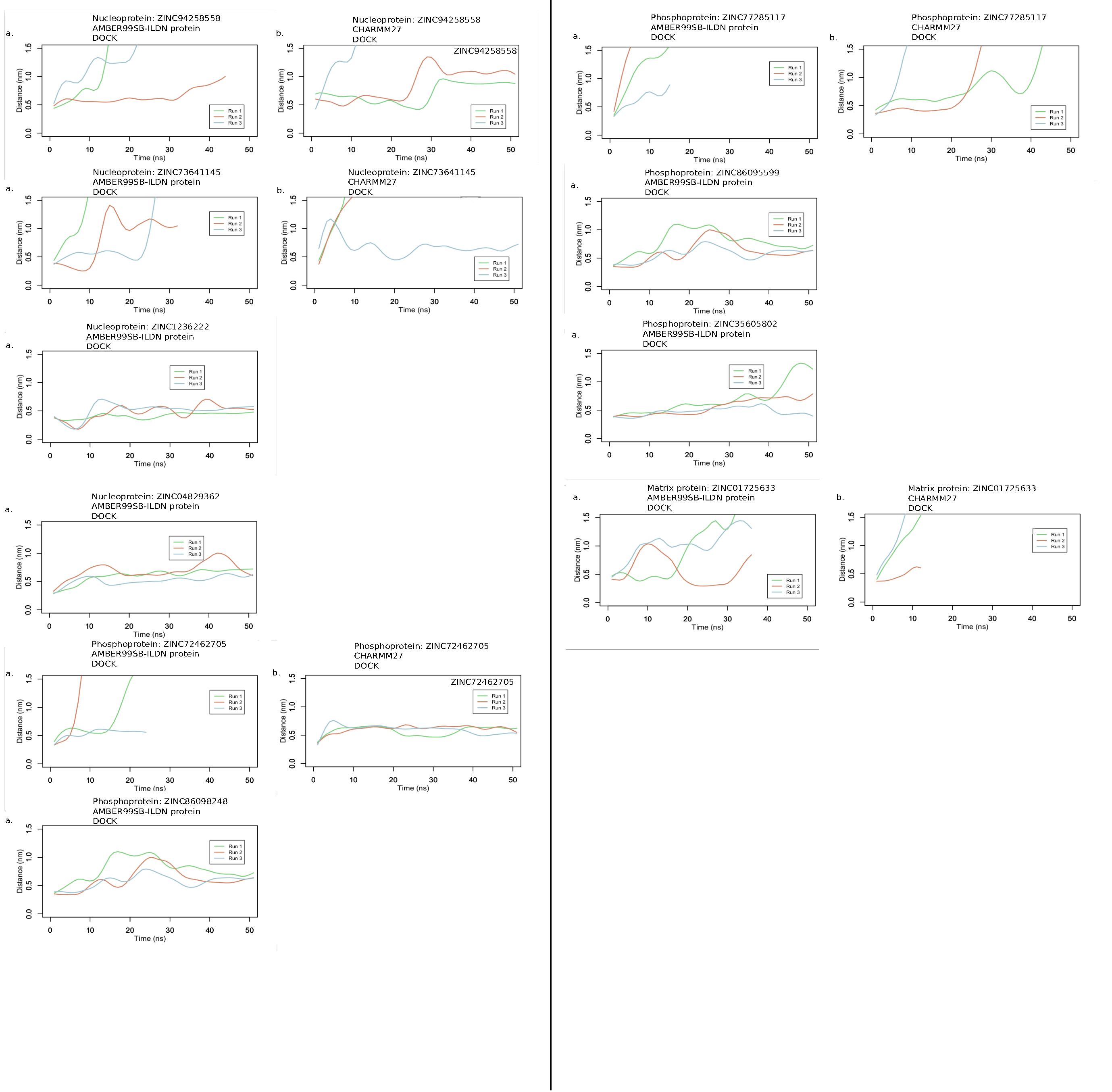

Supplement: S6 Fig — The identity of the ligand, force field and docking strategy used and the target protein has been indicated above each plot. (TIF) [file pntd.0007419.s021.tif]

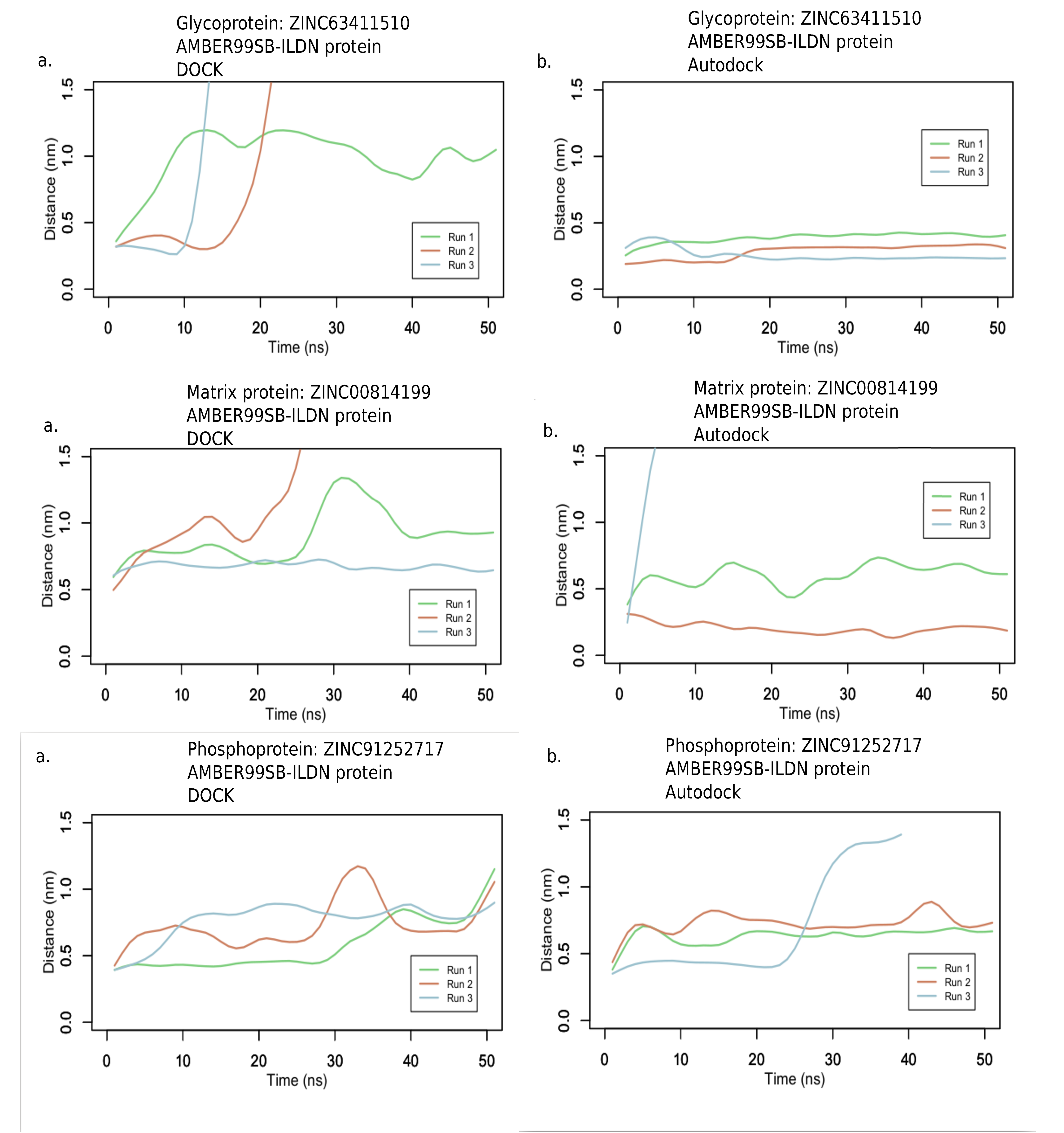

Supplement: S7 Fig — The identity of the ligand, force field and docking strategy used and the target protein has been indicated above each plot. (TIF) [file pntd.0007419.s022.tif]

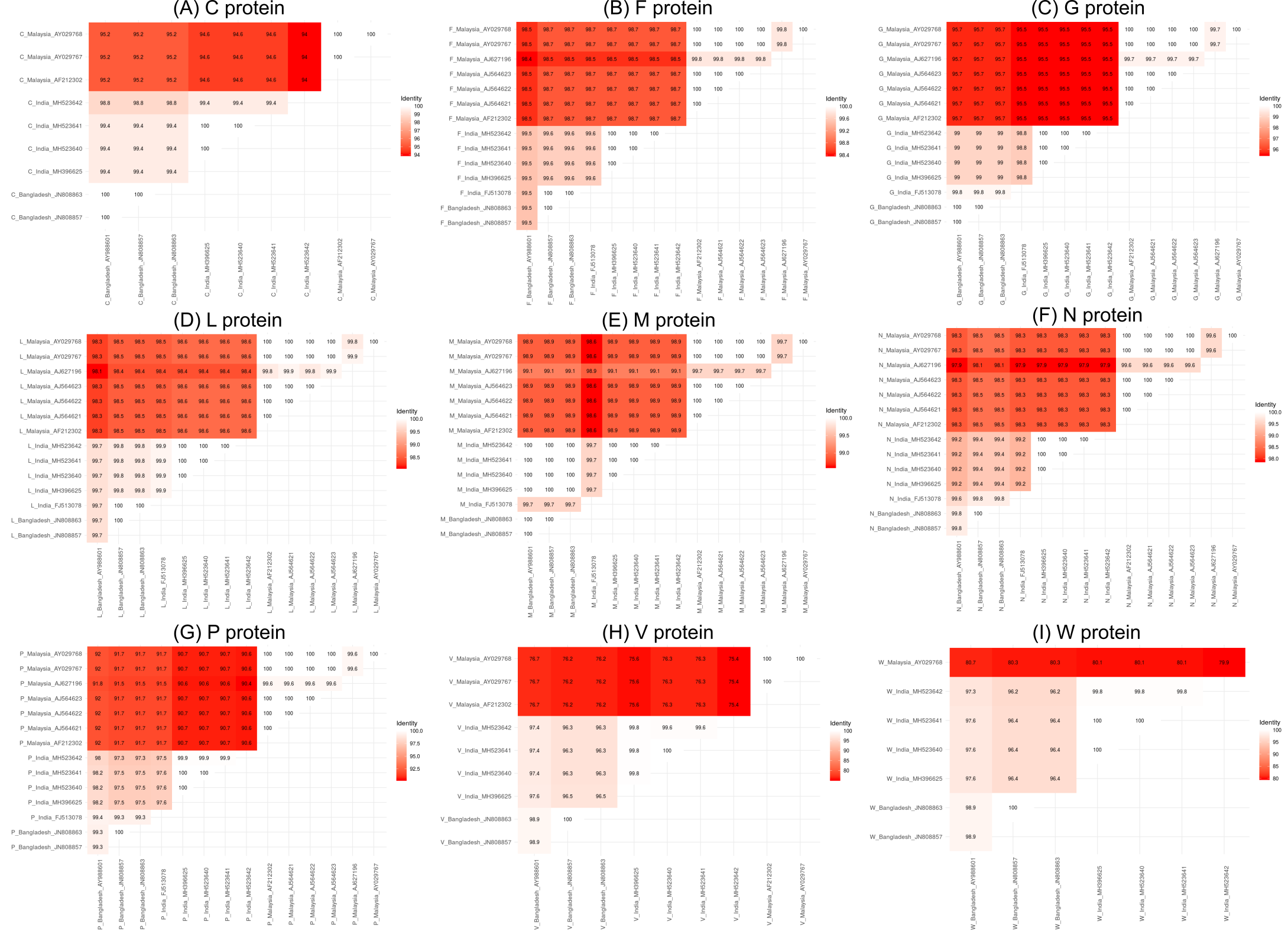

Supplement: S8 Fig — Heatmap showing the sequence conservation between the different strains of NiV for (A) C protein (B) F protein (C) G protein (D) L protein (E) M protein (F) N protein (G) P protein (H) V protein (I) W protein. The color gradient represents sequence conservation where white indicates 100% conservation and redder shades indicate lesser sequence conservation. The labelling convention is Protein_Country_Genome-accession code. (TIF) [file pntd.0007419.s023.tif]

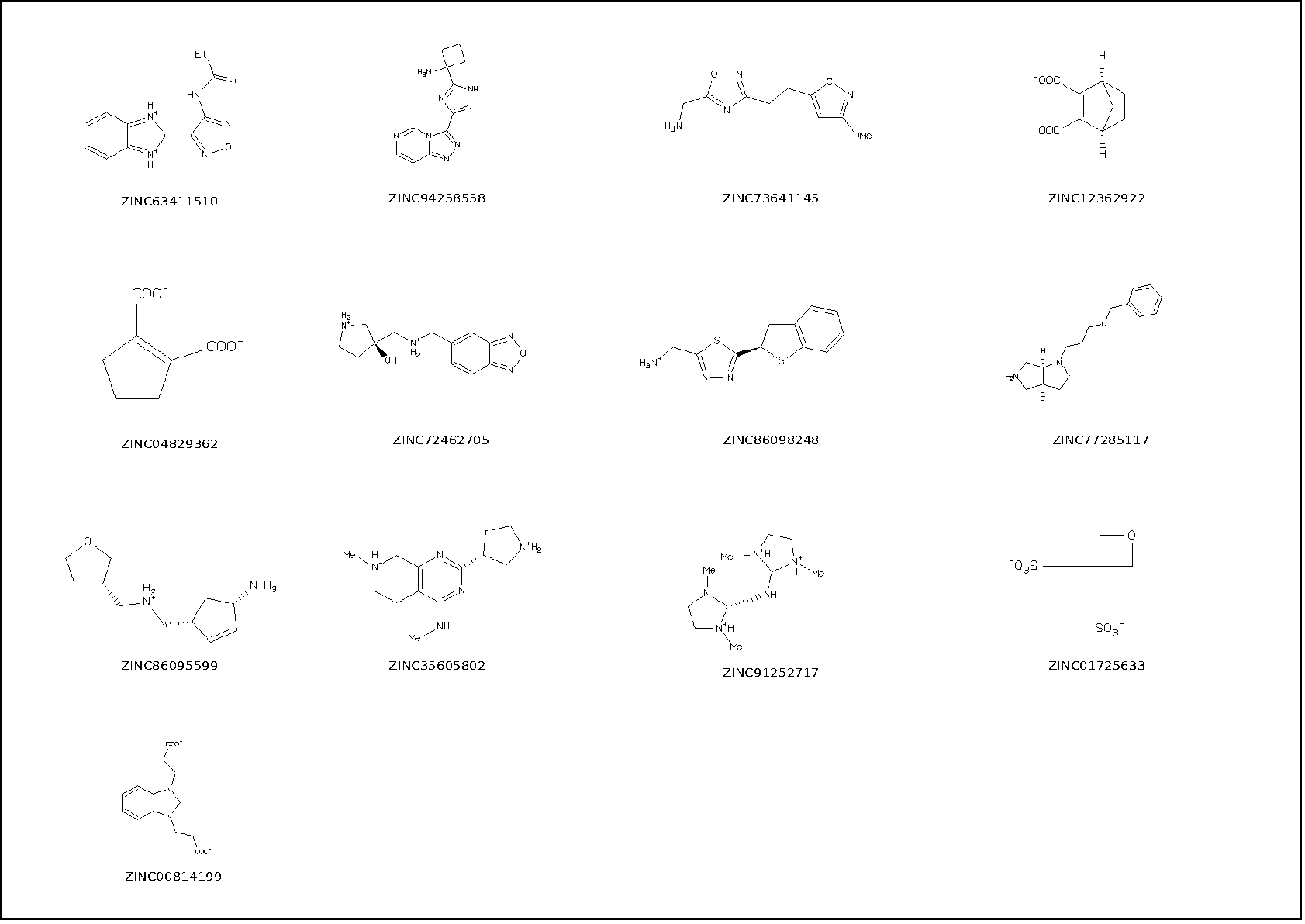

Supplement: S9 Fig — (TIF) [file pntd.0007419.s024.tif]
