# Supplementary material for: Predicting and designing therapeutics against the Nipah virus
Source: PLoS Negl Trop Dis. 2019 Dec 12;13(12):e0007419. doi: 10.1371/journal.pntd.0007419 (PMC6907750; doi:10.1371/journal.pntd.0007419)
Supplement: S1 Text — (DOCX) [file pntd.0007419.s025.docx]

We attempted to model 2 host-pathogen protein complexes involving human cathepsin L with viral F protein and human AP3-B1 with viral M protein. The interacting interfaces of the host-viral complexes are potential targets for designed therapeutics. Human cathepsin-L interaction with F protein is crucial for activation of the F protein to initiate fusion [1]. Viral assembly requires the interaction of M protein with host AP3-B1 [2]. HADDOCK [3], Patchdock [4,5] and Galaxy [6] docking servers were used to model these protein-protein complexes. FoldX, a popularly used and well benchmarked software [8] was used to compute the free energy of complex formation. However, the resulting models had a positive free energy of binding as calculated with FoldX [7], and were therefore not used further in the study. An additional scoring by PIZSA predicted these complexes as non-binders.
